# A Linkable, Polycarbonate Gut Microbiome‐Distal Tumor Chip Platform for Interrogating Cancer Promoting Mechanisms

**DOI:** 10.1002/advs.202309220

**Published:** 2024-07-18

**Authors:** Danielle S.K. Brasino, Sean D. Speese, Kevin Schilling, Carolyn E. Schutt, Michelle C. Barton

**Affiliations:** ^1^ Cancer Early Detection Advanced Research Center, Knight Cancer Institute Oregon Health and Science University Portland OR 97201 USA; ^2^ Department of Biomedical Engineering Oregon Health and Science University Portland OR 97201 USA

**Keywords:** cancer, gut microbiomes, microphysiological systems, organ‐on‐chips

## Abstract

Gut microbiome composition is tied to diseases ranging from arthritis to cancer to depression. However, mechanisms of action are poorly understood, limiting development of relevant therapeutics. Organ‐on‐chip platforms, which model minimal functional units of tissues and can tightly control communication between them, are ideal platforms to study these relationships. Many gut microbiome models are published to date but devices are typically fabricated using oxygen permeable polydimethylsiloxane, requiring interventions to support anaerobic bacteria. To address this challenge, a platform is developed where the chips are fabricated entirely from gas‐impermeable polycarbonate without tapes or gaskets. These chips replicate polarized villus‐like structures of the native tissue. Further, they enable co‐cultures of commensal anaerobic bacteria Blautia coccoides on the surface of gut epithelia for two days within a standard incubator. Another complication of commonly used materials in organ‐on‐chip devices is high ad‐/absorption, limiting applications in high‐resolution microscopy and biomolecule interaction studies. For future communication studies between gut microbiota and distal tumors, an additional polycarbonate chip design is developed to support hydrogel‐embedded tissue culture. These chips enable high‐resolution microscopy with all relevant processing done on‐chip. Designed for facile linking, this platform will make a variety of mechanistic studies possible.

## Graphical abstract

Two linkable organ‐on‐chip platforms are fabricated entirely of polycarbonate to simulate the gut microbiome and distal solid tumors. Leveraging the low oxygen permeability and low ad‐/absorption characteristics of this material, these chips enable co‐culture with anaerobic gut bacteria and high‐resolution on‐chip imaging of tumor constructs.

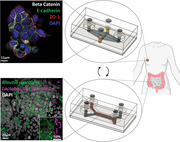

## 1 Introduction

Gut microbiomes have received a dramatic increase in attention for their substantial roles in maintaining human health.^[^ ^1^, ^2^ ^]^ Moreover, diseases ranging from atherosclerosis^[^ ^3^ ^]^ to Alzheimer's^[^ ^4^ ^]^ to cancer^[^ ^5^, ^6^, ^7^ ^]^ have been correlated with changes in gut microbiome composition. These findings can be credited, in large part, to the vast reductions in sequencing costs over recent years. However, mechanisms of action behind these relationships are still poorly understood, leaving unexplored opportunities toward the improvement of targeted healthcare.

While in vitro and in vivo models have enabled the testing of some hypotheses behind gut microbiome‐disease correlations, critical issues in their implementation remain. In vivo models facilitate the study of specific microbial compositions through inoculation of gnotobiotic mice to study host‐microbe interactions.^[^ ^8^ ^]^ However, these systems can suffer from poor translation to patient outcomes due to key differences in diet and physiology.^[^ ^9^ ^]^ Alternatively, in vitro culture can leverage human cells. But these studies also suffer from limitations due to oversimplification using single cell types, culture conditions which differ greatly from native biology, and rapid overgrowth during co‐culture studies.^[^ ^10^ ^]^

Organ‐on‐chip models offer a unique solution toward the study of host‐microbe interactions. These platforms aim to bridge the gap between two‐dimensional cell culture and patient outcomes by using human‐derived cellular material, three‐dimensional growth environments, and mechanical stimuli for the construction of minimal functional units of target tissues.^[^ ^11^ ^]^ With the capacity to connect flow between two or more microphysiological systems (MPS),^[^ ^12^, ^13^ ^]^ as they are also called, it becomes possible to isolate particular inter‐organ relationships.^[^ ^14^, ^15^, ^16^ ^]^ This makes these platforms an optimal model to study mechanisms behind the association between gut microbes and disease arising in non‐adjacent tissue.

Previous work has demonstrated the formation of microbe‐epithelial interfaces for modelling the gut microbiome.^[^ ^17^, ^18^, ^19^, ^20^, ^21^, ^22^ ^]^ These systems utilize flow to drive formation of polarized villus‐like structures with strong barrier function over just a few days. Flow or mechanical deformation also enables extended co‐culture with either predefined bacterial species^[^ ^17^, ^19^, ^20^, ^21^ ^]^ or an inoculum derived from fecal samples to replicate a full microbial community.^[^ ^18^ ^]^ A majority of devices are fabricated from polydimethylsiloxane (PDMS), an elastomer which has high oxygen permeability and good optical properties. Mechanical properties of PDMS have been used to apply cyclic deformation as a way to mimic stimuli such as peristalsis, and oxygen permeability can be leveraged as an oxygen delivery mechanism for dependent cell types. However for gut microbiome recapitulation, this oxygen permeability requires that the system is used within anaerobic chambers or under constant flow of deoxygenated media to maintain the viability of gut bacteria, most of which are sensitive to oxygen.^[^ ^23^ ^]^ Further, PDMS suffers from high levels of absorption and adsorption of molecules into the material and onto the surface, respectively.^[^ ^24^, ^25^, ^26^ ^]^ This characteristic can limit the applications in on‐chip imaging and small molecule treatment studies, and disrupt secreted cell signals. However, application of specific coatings can mitigate this problem.^[^ ^27^ ^]^ To circumvent these issues, some labs have developed chips from gas‐impermeable materials such as polycarbonate.^[^ ^20^ ^]^ However, assembly can be more labor intensive than PDMS device production and often involves the use of silicone gaskets or double‐sided tapes which suffer from similar or worse absorption characteristics.^[^ ^28^, ^29^ ^]^

Much work has also been done in the field of organs‐on‐chip to model disease using spheroids,^[^ ^30^ ^]^ organoids,^[^ ^31^, ^32^ ^]^ and micro‐dissected biopsy tissue.^[^ ^33^ ^]^ This includes extensive development of hydrogel‐embedded culture in order to engineer parenchymal tissue models.^[^ ^34^ ^]^ It is important to note that geometric constraints of complex constructs on‐chip can limit imaging applications requiring thoughtful design.^[^ ^32^ ^]^

**Figure 1:**
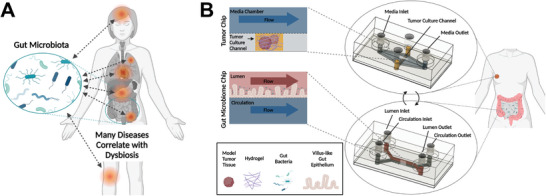
Overview of gut‐microbiome disease interactions and a platform to study mechanisms behind these relationships. A) Illustration of the variety of diseases associated with the gut microbiome including depression, Alzheimer's, cancer, atherosclerosis, diabetes, obesity, Irritable Bowel Disease (IBD), arthritis, and more; B) Illustration of the tumor chip and gut microbiome chip platforms presented herein, designed for studies into gut microbiome‐tumor associations.

Herein, we report the development and application of MPS devices composed solely of polycarbonate. This material facilitates control over oxygen exposure to culture gut microbiome interface models using standard tissue culture infrastructure. Further, tumor chip devices support culture of a variety of hydrogel embedded tissue models. Polycarbonate's material properties optimize the system for future applications in multi‐organ studies to understand mechanisms behind gut dysbiosis and cancer progression (**Figure** **1**).

## 2 Results and Discussion

### 2.1 Fabrication of All‐Polycarbonate Chip Platforms

Most MPS platforms leverage soft lithography techniques to generate chips from PDMS due to the rapid production capabilities of this method. However, this comes with the cost of high ad‐/absorption characteristics, affecting the transport and delivery of molecules based upon their hydrophobicity.^[^ ^35^ ^]^ Additionally, PDMS is highly oxygen permeable, requiring added infrastructure or methodologies to culture anaerobic species.^[^ ^18^, ^21^ ^]^ To circumvent these issues, chips were developed entirely from a thermoplastic polymer, polycarbonate. While others have leveraged thermoplastics in this regard, plastics were interfaced using double‐sided tapes^[^ ^28^ ^]^ or channels were introduced using silicone rubber gaskets.^[^ ^20^ ^]^ These materials also suffer from high absorption characteristics^[^ ^29^ ^]^ and introduce multiple material interfaces for cell growth which could have negative implications when trying to culture a continuous epithelial or endothelial tissue.

In order to generate a fully polycarbonate device, all channel components were modeled using Autodesk manufacturing software, Fusion 360 (Figure S1, Supporting Information), followed by precision milling using a computer numerical control (CNC) micro‐milling machine. This process supports the rapid design and prototyping of new chip geometries. Further, this process eliminated use of hazardous piranha or hydrofluoric acid etching agents critical to photolithography. Flexibility afforded by these manufacturing processes enabled many iterations on device design both in broad scale geometries and fine tuning of component dimensions.

**Figure 2:**
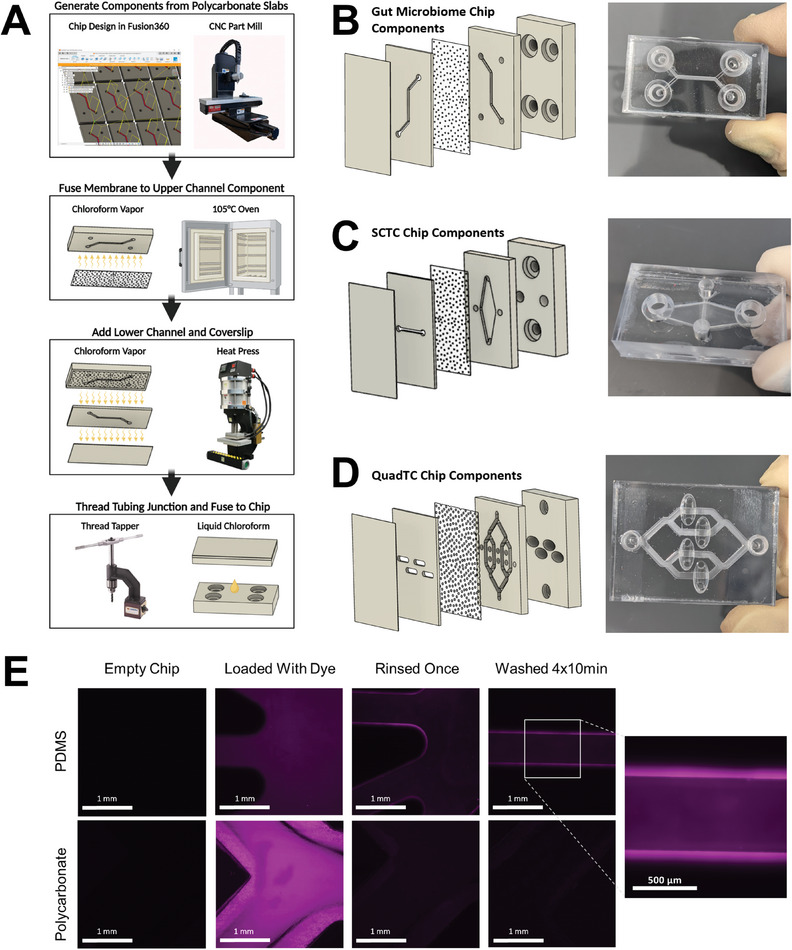
Fabrication of polycarbonate devices using machine milling and chloroform‐assisted bonding. A) Manufacturing process of polycarbonate chips. Polycarbonate slabs are milled using a Computer Numerical Control (CNC) milling machine and tubing interfaces are adapted for fittings using a manual thread tapper. Components are assembled using vapor or liquid chloroform deposition followed by application of heat and/or pressure. CNC, heat press and thread tapper images are reproduced from Minitech Machinery Corp., Rosin Technologies LLC. and Vertex Machinery Work Co., respectively; B) Gut chip layers and fully assembled gut chip; C) Single channel tumor chip (SCTC) layers and assembled SCTC chip; D) Quad channel tumor chip (QuadTC) layers and assembled QuadTC; E) Absorption and adsorption by PDMS and polycarbonate following 2 h dye incubation and washing by PBS. Strong residual fluorescence only on PDMS channel surfaces.

To generate fully polycarbonate devices, it is necessary to bond the independent components. Common methods for thermoplastic bonding include heating of the component parts to the plastic's melting point or application of relevant solvents to the part surfaces prior to joining. However, these methodologies are unsatisfactory in the generation of microfluidics as they can severely impact the channel morphology. Vapors of appropriate solvents can swell a thin layer of the thermoplastic surface. Exposure to solvent vapor followed by compression at a temperature far below the melting temperature bonds parts without impacting channel geometries^[^ ^36^, ^37^ ^]^ (Figure S2, Supporting Information). This technique was applied to the chip system in two steps to minimize warping of the thin, porous membrane which was sourced commercially from Sterlitech. A thick polycarbonate slab was threaded for tubing interfaces and bonded to the microfluidic chips using traditional solvent bonding for durability during hand‐tightened tubing installation (**Figure** **2**A). The resulting chips could withstand fluid pressures far beyond flow rates used in organ‐on‐chip culture. Gut microbiome chips were tested by flowing water simultaneously through both sides at a rate of 10 mL over 15 s without leaking. Taken together, this multi‐step fabrication process enabled the assembly of gut microbiome (Figure 2B) and tumor chips (Figure 2C,D) made entirely of polycarbonate. By constructing chips from polycarbonate, issues with ab‐/adsorption were reduced compared to channels fabricated in PDMS (Figure 2E).

### 2.2 Polycarbonate Gut Microbiome MPS Model

Gut microbiome model devices described herein use the stacked channel architecture developed by previous epithelial models^[^ ^17^ ^]^ (Figure 1B). These upper and lower chambers replicate the gut lumen and adjacent capillaries, respectively. Following the transition to all‐polycarbonate interfaces, it was necessary to evaluate the extracellular matrix deposition process critical to facilitate cell attachment to the porous membrane surface. Assessment using cell seeding assays and two‐photon microscopy indicated best membrane functionalization when pure collagen was deposited at room temperature in phosphate buffered saline (PBS) overnight, evidenced by cellular monolayer coverage and collagen fibril deposition, respectively, (Figure S3, Supporting Information).

**Figure 3:**
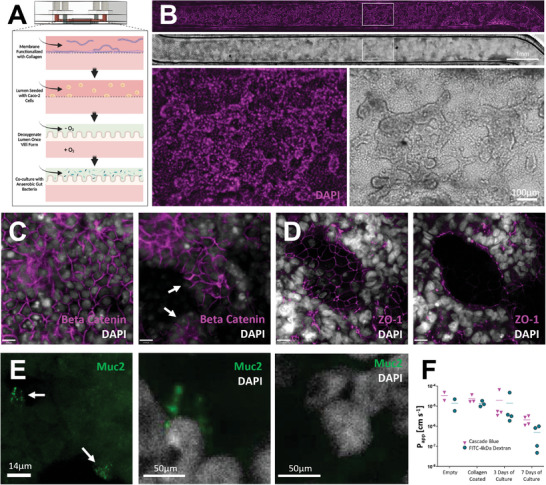
Characterization of polycarbonate gut chips. A) Schematic overview of gut microbiome assembly on chip. Channels are functionalized with collagen prior to Caco‐2 cell seeding. Following growth of villus‐like structures, gut lumen channel media is depleted of oxygen for co‐culture with anaerobic gut microbes; B) Gut chip channel with Caco‐2 cells grown into villus‐like structures, shown in brightfield and with nuclei stained by DAPI (magenta), scale bar denotes 1 mm; C) Immunofluorescence imaging of polarized Caco‐2 layers on day 7 using markers DAPI (greyscale) and anti‐Beta Catenin (magenta) show basolateral staining of beta catenin at intercellular junctions (left) and no staining at apical surfaces (right). Arrows indicate examples of apical surfaces devoid of beta catenin staining, scale bars denote 14 µm; D) Caco‐2 barriers characterized by immunofluorescence imaging of tight junction protein ZO‐1 (magenta) and DAPI (grayscale), shown here on day 9, show ZO‐1 expression at cellular interfaces (left) and apical to nuclei at the villus surface (right), scale bars denote 14 µm; E) A subset of Caco‐2 cells transdifferentiate into goblet‐like cells producing mucin, noted by arrows, shown here on day 9 (left), a pullout shows a cell staining positively for Muc2 (middle), cells without Muc2 staining (right); F) Permeability data of gut chips from initial assembly through seven days of culture tracking Cascade Blue hydrazide and FITC‐dextran 4 kDa.

Following channel functionalization, cells from the colon epithelial cell line CaCo‐2 were cultured in the upper, luminal compartment of the gut chip (**Figure** **3**A). Constant flow of culture media at a rate to match shear strain experienced in the colon^[^ ^38^ ^]^ produced villus‐like structures (Figure 3B) over the course of 3–6 days. To characterize polarization of cells within these constructs, immunofluorescence imaging was employed. By the seventh day of culture, markers of polarization were evident. Staining at basolateral interfaces by anti‐beta catenin indicated that the apical surface was appropriately directed toward the upper, gut lumen compartment (Figure 3C; Figure S4 Supporting Information). Additionally, staining for marker zonula occludens‐1 (ZO‐1) indicated the formation of tight junctions at the intercellular interfaces, critical to the proper function of barrier tissues like the gut epithelium, expressed particularly at the interface of the lumen and closer to the apical end of the cell than the nucleus, indicative of polarization (Figure 3D; Figure S4 Supporting Information). Beyond morphological features of the gut model, an essential component of the microbial niche is the extracellular mucin layer produced by specialized goblet cells. Transdifferentiation of Caco‐2 cells into a goblet cell‐like phenotype is indicated by immunofluorescence staining of the protein mucin 2. Staining of intracellular granule structures, evident as bright spots above background (staining highlighted by arrows), within this subset of cells indicated pre‐secreted mucin (Figure 3E). Functional barrier integrity was assessed via permeability assay. Two dyes of differing size, Cascade Blue hydrazide and FITC‐conjugated dextran 4 kDa, were administered to the lumen compartment and flux into the circulation compartment was measured in collected effluent. In chips fabricated with a 1 µm pore size membrane, permeability decreased in the majority of chips following three days of culture as cell layers became confluent. By day seven, P_app_ reached 2.1E‐06 cm s^−1^ for Cascade Blue hydrazide and 4.9E‐07 cm s^−1^ for FITC‐conjugated dextran (Figure 3F). Four kDa dextran conjugated with FITC was used, as smaller dextrans better assess barrier integrity and this size is utilized for permeability measurements in vivo.^[^ ^39^ ^]^ Compared to colon tissue explants maintained within a microfluidic device, our system demonstrates stronger barrier function with permeability values half previously reported values for such devices.^[^ ^40^ ^]^ Meanwhile the permeability for Cascade Blue hydrazide was a few times greater than reported values of tight barrier formation in primary tissue‐derived Colon‐on‐Chip devices,^[^ ^41^ ^]^ suggesting room for improvement potentially through integration of more diverse cell types.

**Figure 4:**
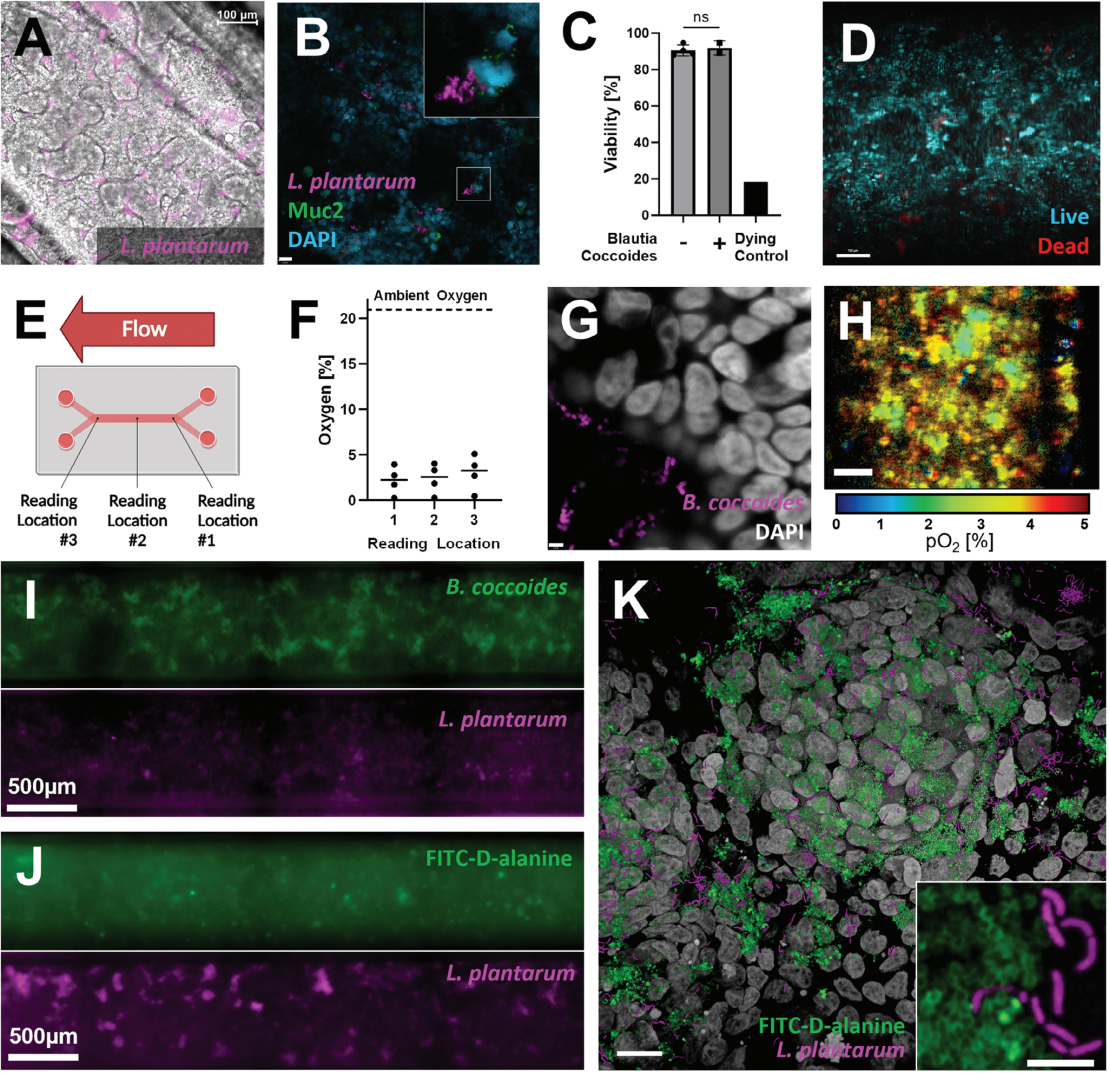
Further characterization of gut chips co‐cultured with facultative and obligate anaerobic bacteria. A) Overlaid brightfield and immunofluorescence image of Caco‐2 villi co‐cultured with mCherry expressing *Lactobacillus plantarum*, scale bar denotes 100 µm; B) Immunofluorescence image of Caco‐2 cell nuclei (cyan), *Lactobacillus plantarum* (magenta), and mucin granules produced by goblet‐like cells (green), scale bar denotes 21 µm; C) Gut chip viability assessment validates low cell death upon exposure to luminal oxygen depletion and no reduction in viability in co‐culture with commensal anaerobe *Blautia coccoides*, one‐way ANOVA with a Bonferroni post hoc, error bar denotes standard deviation, *n* = 2–4 chips; D) Representative image of 9‐day old gut chips cultured with low luminal oxygen showing live (blue) and dead cells (red), scale bar denotes 100 µm; E) Schematic depicting oxygen reading locations taken along the overlapping region within the gut chip; F) Oxygen readings taken within the lumen of a 9‐day old gut chip show an average 2–3% oxygen along the length of the chip; G) Immunofluorescence image of *B. coccoides* cocultured within a gut chip for two days. Bacteria integrated FITC‐D‐alanine (magenta) into cell walls during the second day of culture and Caco‐2 nuclei were stained with DAPI (greyscale), scale bar denotes 3.5 µm. H) Phosphorescence lifetime image of oxygen tension (pO_2_) in a gut chip grown for ten days with three days of co‐culture with *B. coccoides* showing pO_2_ within the gut villus‐like architecture consistent with an average of 4% oxygen, scale bar denotes 100 µm. I) Fluorescence image with split channels showing *B. coccoides* pre‐labelled with FITC‐D‐alanine and *L. plantarum* expressing mCherry 2.5 h after inoculation on‐chip. J) Fluorescence image with split channels showing *B. coccoides* and *L. plantarum* expressing mCherry after 48 hours on‐chip and following overnight labelling with FITC‐D‐alanine. K) Three‐dimensional rendering of fluorescence images showing *B. coccoides* labelled with FITC‐D‐alanine (green) and *L. plantarum* expressing mCherry (magenta) co‐cultured on a CaCo‐2 surface for 45 h, genetic material labelled with DAPI (grayscale), scale bar denotes 20 µm. Pull‐out depicts a single z‐plane without DAPI channel, highlighting distinct bacterial morphologies, scale bar denotes 5 µm.

Following growth of the villus‐like gut epithelium, a microbial community may be introduced to the gut lumen to simulate the gut microbiome. For any bacteria of interest tolerant to oxygen, such as the commensal bacteria *Lactobacillus plantarum*, chips may be inoculated immediately following villus growth. By utilizing a strain genetically engineered to express the fluorescent protein mCherry, bacteria could be tracked using fluorescence microscopy. Bacteria were found to reside within inter‐villus spaces (**Figure** **4**A). Commensal bacteria, including several *Lactobacillus* species, have been shown to enhance expression of gut‐protective mucins.^[^ ^42^, ^43^ ^]^ Immunofluorescence imaging of co‐cultures within our platform demonstrates large numbers of mucin granules within goblet‐like cells alongside mCherry expressing *L. plantarum* (Figure 4B).

The vast majority of gut bacteria are obligate anaerobes, many intolerant to any handling done at the bench. To culture these bacteria, all handling must be done under an inert atmosphere devoid of oxygen. Gut epithelia, on the contrary, require oxygen. To replicate the gut microbiome interface, it became necessary to provide an oxygen‐free environment for the bacterial compartment, while delivering oxygen to any mammalian cell culture in a simulation of the enteric circulatory system. By utilizing oxygen impermeable materials, our platform enables maintenance of an anaerobic condition following removal of oxygen from the media reservoir. To simulate the gut microbiome interface, media reservoirs for the lumen compartment were depleted of oxygen following growth of gut villi, while lower channel media supplied oxygen to Caco‐2 cells (Figure 3A). Assessments following oxygen depletion show Caco‐2 cell layers are over 80% viable (Figure 4C,D). Using OXNANO probes dispersed in the media supplied to the lumen compartment, an optical sensor detected bulk oxygen concentrations of 2–3% on average, which is within physiological ranges^[^ ^44^ ^]^ (Figure 4E,F; Figure S6 Supporting Information). Under the microaerophilic conditions of the colon, diverse microbial communities depend upon a range of oxygen microenvironments.^[^ ^45^ ^]^ Using phosphorescence lifetime imaging to assay the microenvironmental oxygen tension across the cell surface, a range of measured oxygen tension averaging ≈4% could be discerned (Figure 4H). Additional HIF1α staining was performed and showed no clear increase in nuclear signal within the CaCo‐2 cells compared to hypoxic 2D controls. Altogether, this is concordant with the steep oxygen gradient between the ambient oxygen levels delivered by the circulatory compartment and the anaerobic environment of the gut lumen, simulating gradients that exist in human tissues and demonstrating future capabilities of the chip to support full microbial consortia.

To demonstrate capability of co‐culture with obligate anaerobes, gut lumens in chips fabricated with 0.2 µm pore size membranes were inoculated with the commensal bacteria, *Blautia coccoides*. Following two days of co‐culture, media was collected from each chamber and plated under anaerobic conditions to assay presence of viable bacteria. Effluent from the lumen compartment grew substantial colonies while effluent from the lower chamber did not (Figure S7, Supporting Information). Epithelia showed no reduction in viability in the presence of bacteria (Figure 4C; Figure S5 Supporting Information). These results successfully demonstrate longitudinal co‐culture of two biological components with discrete culture requirements to recapitulate the gut microbiome interface. To assay metabolic activity of the bacteria on‐chip, fluorescently conjugated D‐alanine was added to the culture media after a day of co‐culture. Following a day of culture in the presence of fluorescent D‐alanine, microscopy indicates substantial uptake of the molecule and integration into the bacterial cell wall. This enables on‐chip tracking of the bacteria, which reside at the villus surface and in the intervillous space, and demonstrates their continued metabolic activity while on‐chip (Figure 4G; and Video S1, Supporting Information).

Microbiomes are composed of many microbial taxa. Complex communities may be reconstituted from patient‐derived material or model consortia may be constructed from pre‐selected species. To establish the ability to culture multiple species at once on‐chip, a mixture of *L. plantarum* expressing mCherry and *B. coccoides* grown with fluorescently conjugated D‐alanine were inoculated. Following 2.5 h on‐chip, both species could be identified (Figure 4I). Prior to imaging at 48 h of culture on‐chip, bacteria were cultured with fluorescently conjugated D‐alanine overnight. Fluorescently conjugated D‐alanine appeared to be preferentially taken up by *B. coccoides* indicated by a lack of colocalization between mCherry and dye signal. This may be explained by selectivity for D‐lactate over D‐alanine in *L. plantarum*.^[^ ^46^ ^]^ In combination with detection of *L. plantarum* via mCherry expression, this enabled detection of bacteria colonies after two days of co‐culture (Figure 4J,K) demonstrating the ability to culture multiple bacteria with differing oxygen tolerances together on‐chip.

To quantify bacteria load, three gut chips were inoculated with *L. plantarum* and *B. coccoides* and grown for two days with introduction of FITC‐D‐alanine to the luminal media reservoir at the end of day one. CFU counts from luminal media collected at 45 h averaged 2.5 × 10^8^ consistent with bacterial densities found in the ileum.^[^ ^47^ ^]^ Plating of media from the circulatory chamber showed 0, 72, and 1377 CFU across the three devices, suggesting the need to improve production of the dense, protective mucus layer. Bacteria adhered to the CaCo‐2 surface were fixed in place and the membrane was extracted for high‐resolution microscopy. Resulting images depict the FITC‐labelled cell wall of *B. coccoides* and the mCherry labeled *L. plantarum*, highlighting the distinct differences in cellular morphology between the two species (Figure 4K).

### 2.3 Polycarbonate Tumor Chip Model for Hydrogel‐Embedded Culture

In vitro systems, MPS included, involve a reduction in complexity to model tissues of interest. To target mechanisms of interest or optimize outcomes for patients using personalized medicine, the degree to which complexity is reduced varies. Based upon experimental constraints, different tissue models are employed ranging from cell line‐derived spheroids to patient‐derived tissue. To optimize utility of our device in future applications leveraging these varied model tissues, our system incorporates hydrogel‐embedded culture with nutrient and waste flux via unidirectional fluid flow. The high degree of absorption and adsorption inherent in materials like PDMS^[^ ^24^, ^25^, ^26^ ^]^ commonly used for culture platforms limits applications in drug dosing studies and analytical techniques such as imaging. By constructing platforms using our polycarbonate chip fabrication protocol, we optimized our system for a wide variety of applications by reducing ab‐/adsorption issues. Specifically, engineering the platform for high‐resolution imaging on‐chip makes it possible to conduct long‐term live‐cell imaging to study characteristics at a range of timescales. Using cancer cell line‐derived spheroids or patient‐derived organoids, this platform will facilitate studies into the relationship of gut microbiome composition and distal solid tumors in organs such as the breast.^[^ ^7^ ^]^

**Figure 5:**
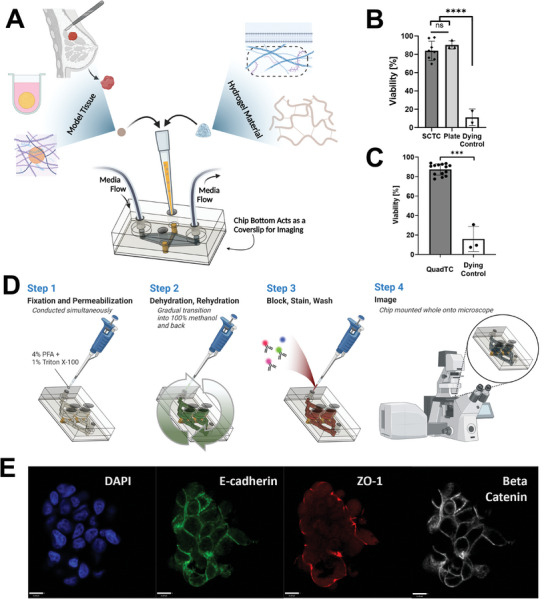
Characterization of tumor chips capable of high‐resolution on‐chip imaging. A) Schematic overview of tumor chip capabilities. Tissue channels may be loaded with a range of materials including spheroids and organoids, extracellular matrix proteins, and synthetic polymers. Devices are designed for longitudinal studies including on‐chip imaging; B) SCTC following two days of on‐chip culture in 1.45 mg/mL collagen maintain equivalent viability to spheroids maintained in liquid culture, ****p < 0.0001, one‐way ANOVA, error bar denotes standard deviation, taken from three chips and three plate spheroids; C) High viability of spheroids cultured in 2.7 mg mL^−1^ collagen in QuadTC for two days, ***p < 0.005, Student‘s *t*‐test, error bar denotes standard deviation, *n* = 1–3 spheroids per well, across 4 wells; D) Schematic of on‐chip staining protocol for immunofluorescence imaging; E) Confocal microscopy image of MCF7 spheroid loaded with collagen in QuadTC chip. Stained for DAPI (blue), E‐cadherin (green), ZO‐1 (red), and Beta‐Catenin (grayscale), scale bar represents 11 µm.

Model tissues and support matrices are loaded into a recessed channel while independent ports for tubing feed a diamond shaped channel for media (**Figure** **5**A). Hydrogel and media compartments of the single‐channel tumor chip (SCTC) are separated by a porous membrane to avoid occlusion of flow. Targeting flow perpendicular to the tissue culture channel limits accumulation of excreted factors from treated tissues. This is an issue of previous model chips that culture tissues in series as it results in heterogenous culture conditions.

By using polycarbonate it becomes possible to tightly control nutrient and oxygen flux due to the reduced permeability of polycarbonate. To ensure this reduced oxygen influx did not negatively impact tissue viability, spheroids comprised of MCF7, a breast cancer cell line, were cultured in SCTC embedded in a collagen matrix for two or three days. Staining with Hoechst 33342 and ethidium homodimer established no reduction in viability compared to spheroids maintained in liquid culture in low adhesion plates (Figure 5B; Figures S8 and S9 Supporting Information). While spheroids make many mechanistic studies possible, personalized medicine requires the use of patient‐derived material either as whole tissue or dissociated and developed into self‐assembled organoids. To test these more complex models, a patient‐derived kidney cancer organoid line was cultured in basement membrane extract within the SCTC for one week. These organoids also maintained high viability (Figure S10, Supporting Information).

A second chip design, QuadTC, cultures four, independent recessed hydrogel channels, expanding upon the tumor chip capabilities by enabling replicates within a single chip (Figure 2D). Hoescht 33342 and ethidium homodimer staining confirm high viability across the channels of MCF7 cell line‐derived spheroids embedded in collagen (Figure 5C; Figure S11 Supporting Information).

Because model tissues are cultured adjacent to the coverslip bottom and polycarbonate does not suffer from high fluorophore or biomolecule adsorption, staining and high‐resolution imaging can be done on‐chip. Tissue processing protocols included simultaneous fixation and treatment to permeabilize membranes followed by methanol dehydration and rehydration in phosphate buffered saline. These techniques improved imaging at depth in dense spheroids. Taken together these techniques, which can all be done on‐chip due to compatibility with our chip geometry, enable acquisition of high‐resolution microscopy data without removal from the chip, avoiding processing which could damage tissues or alter the microenvironment (Figure 5D). As a demonstration of function, staining showed polarity of spheroids loaded into a QuadTC (Figure 5E) or SCTC (Figure S12, Supporting Information).

### 2.4 Linked Gut Microbiome‐Tumor Chip Culture for Distal Interaction Studies

Patient sequencing studies have shown a correlation between gut microbiome composition and a wide range of disease states, including several cancer subtypes. However, the mechanistic underpinnings of these correlations are poorly understood. Connecting media flow between organ‐on‐chip devices offers a means to assess hypotheses and interrogate specific mechanisms through which disease at distal organ sites could be impacted by gut microbes.

**Figure 6:**
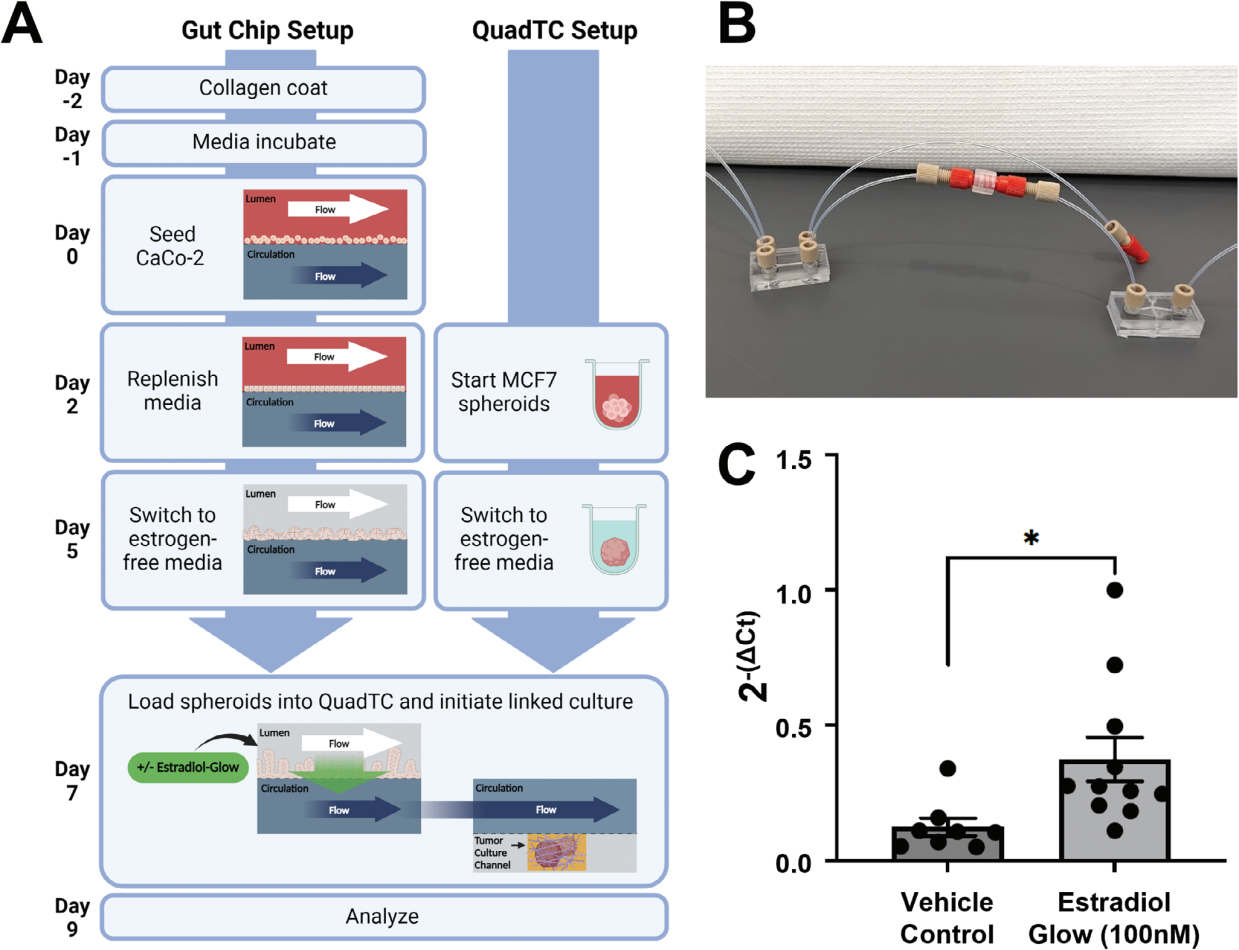
Demonstration of gut chip and QuadTC linked culture using estrogen as a model. A) Schematic overview of linked culture experiments. Gut chips are cultured to form villus structures followed by removal of estrogen from inlet media. ER+, MCF7 spheroids are grown for three days and then transitioned to estrogen‐free media. At day 7 of gut chip culture, spheroids are loaded into QuadTC and connected to gut chip circulatory effluent. Gut chips may be fed with model molecules, such as a fluorescently labeled estradiol, through the lumen compartment to model absorption and circulation of metabolites. B) Example of simple tubing interconnects to enable linked culture using a gut chip (left) and SCTC (right). C) Following two days of linked culture, significantly increased expression of GREB1, an estrogen‐responsive gene transcript, was detected in spheroids downstream of gut chips fed a fluorescently labeled estradiol, Estradiol Glow, *, *p* = 0.013, Welch's *t*‐test, *n* = 8–11 over two experiments.

Using the same fittings which connect tubing to our polycarbonate devices and syringes, flow can be shared across multiple chips (**Figure** **6**B). By directing flow from the circulatory compartment of the gut microbiome chip to tumor chips, it becomes possible to demonstrate absorption and circulation of metabolites. To demonstrate this function in our linked chip system, estrogen was selected as a model molecule as it is a hypothesized driver of gut microbe‐breast cancer correlations.^[^ ^48^ ^]^ To eliminate the estrogenic activity caused by commonly used tissue culture media components, such as phenol red, gut chips and ER+ MCF7 spheroids were transitioned to an estrogen‐free media two days prior to initiation of linked culture. Using QuadTC chips, several biological replicates of MCF7 spheroids could be assayed for response to gut chip effluent. These devices were connected inline to gut chip circulatory compartments and lumen inlet media was fed with estrogen‐free media supplemented with a fluorescent‐labelled estradiol (100 nм) or ethanol vehicle control (0.01% v v^−1^) (Figure 6A). Following 44–48 h of linked culture, a subset of devices were stained with Hoechst and imaged via confocal microscope. Gut chips demonstrated clear staining with the fluorescent‐labeled estradiol, however no signal could be detected in the QuadTC, likely due to limitations in sensitivity of imaging to see low levels of the Estradiol Glow. For higher sensitivity detection of estrogen signaling, spheroids and gut chips were collected for RNA extraction and qPCR assay of an estrogen‐responsive gene transcript, *GREB1*. Gut chips did not exhibit increased *GREB1* expression, which was unsurprising since *GREB1* is enriched for expression in hormone‐responsive tissues.^[^ ^49^ ^]^ In contrast, MCF7 spheroids downstream of gut chips fed with Estradiol‐Glow supplemented media had significantly increased expression of *GREB1* versus vehicle control‐treated samples (Figure 6C).

These results demonstrate the capabilities of our two‐chip platform for biologically relevant communication with regulatory impact, as a model of gut microbiome influence on distal tissues and tumors, such as the breast. Identification of changes to RNA expression resulting from uptake of estrogen across the gut epithelium and delivery through the circulation illustrate the potential of the system to study absorption of metabolites of interest and their delivery to downstream tumor constructs. While localization of Estradiol Glow was outside the limits of detection for microscopy, it was possible to track phenotypic changes induced by metabolite signaling to better understand microbiome‐driven disease progression.

## 3 Conclusion

This work demonstrates the capabilities of an all‐polycarbonate organ‐on‐chip platform. By leveraging organ‐on‐chip design concepts from the literature, modified to use an oxygen‐impermeable, low‐binding material permissive of fluorescence microscopy, these devices maximize analytical capabilities while expanding culture options. Using vapor‐assisted fusing, milled components were assembled without the use of tapes or gaskets, minimizing ab‐/adsorption issues typically found in PDMS and other commonly used materials. Two‐channel epithelial chip models replicated villus structure growth from Caco‐2 cells. With reduced oxygen permeability, polycarbonate facilitated co‐culture of an obligate anaerobe on the surface of gut epithelial cells and structures without specialized incubation conditions. To begin simulating a gut microbiome interface, two bacterial species with different environmental requirements were cultured together in the gut chip for two days. Additionally, to enable modeling of gut microbiome interactions with distal tumors, polycarbonate chips for culture of hydrogel‐embedded models were developed. These tumor‐on‐chip devices supported viability of cell line‐derived spheroids and patient‐derived organoids. The material properties and culture geometry of these devices offer capabilities for numerous analytical readouts with a particular focus on on‐chip processing and imaging for high‐resolution fluorescence microscopy. These properties enable long‐term live‐cell imaging experiments to study a continuum of phenotypic changes, instead of relying on terminal experiment analyses.

RNA retrieval was achieved simply from the devices for analyses, such as qRT‐PCR. Specifically, hydrogel‐embedded constructs may be retrieved by removal of the entire hydrogel for RNA extraction off‐chip after chip layers are separated. For gut microbiome chips, RNA was acquired after gut cells were lifted with brief application of TrypLE dissociation reagent or by direct RNA extraction on‐chip. As a demonstration of this two‐device system in simulating metabolite uptake and regulatory communication between distal tissues, gut chips were treated with a fluorescently‐labelled estradiol. Treatment led to a significant increase in estrogen‐responsive gene expression within spheroids connected to the gut circulatory compartment.

The polycarbonate chips enable anaerobic culture of gut microbiomes, which are composed predominantly of oxygen‐sensitive microbes. Further, because human gut epithelial cells are supported by delivery of oxygen through a secondary media channel, this platform enables direct connection with tumor‐on‐chip devices for signaling studies with other oxygen‐dependent tissues while maintaining the gut microbiome interface. By eliminating absorption and adsorption issues, biomolecules can flow freely between the two chips. With flow driven unidirectionally, this enables direct study of the effect one tissue has on another.

Due to the flexibility built into this culture system, our platform could be applied in the future to a wide range of diseases associated with gut dysbiosis. Single and quad‐channel tumor chips supporting hydrogel‐embedded culture can be leveraged with a vast array of tissue‐ or disease‐specific spheroids and organoids to model a diverse range of diseases. In addition, similarities to biopsy culture devices suggest even wider options in selecting model tissues. These disease models can then be paired with gut microbiome chips to target study of particular gut microbiome compositions, microbial metabolites, drug metabolism, and more. Complexity may be expanded further in the future by incorporation of cell types such as peripheral blood mononuclear cells for studies into potential mechanisms driving tumor progression and its association with the gut microbiome.

Using simple tubing interconnects, gut and disease tissue models can be grown independently to reach optimal growth stages prior to initiation of linked culture. If early establishment of disease tissue is not necessary, future devices could integrate both geometries into a single chip to minimize dead volume. Environmental conditions such as pH and nutrient availability can be easily analyzed in collected effluent. Alternatively, conditions could be studied longitudinally by integrating sensors.^[^ ^50^ ^]^ Altogether, this novel chip platform will empower mechanistic studies into gut microbiome‐disease correlations to aid in improved intervention strategies and better patient outcomes.

## 4 Experimental Section

### Materials

Caco‐2 cells were acquired from Emulate Inc. with purchase of an Emulate Bio‐kit. All other materials were sourced from standard vendors including; polycarbonate (McMaster‐Carr), porous membranes (Sterlitech), and *Blautia coccoides* (ATCC 29236). *Lactobacillus plantarum* expressing mCherry were a kind gift from Dr. Michael Brasino and MCF7 cells were a kind gift from Dr. Hisham Mohammed. Organoids and organoid culture materials were a kind gift from Dr. George Thomas. Bulk oxygen tension measurements were conducted using OXNANO oxygen nanoprobes and sensor developed by PyroScience. Estradiol Glow was purchased from Jena Bioscience.

### Cell Culture

Mammalian cells were maintained prior to use in standard tissue culture flasks. Caco‐2 cells were cultured in Gibco Dulbecco's Modified Eagle Medium (DMEM) supplemented with fetal bovine serum (FBS) (20%) and penicillin/streptomycin (1%). For gut chip seeding, cells were used at passage 13–18 as noted upon receipt from Emulate, however they note initial passage was unknown. MCF7 cells were cultured in DMEM supplemented with FBS (10%) and penicillin/streptomycin (1%). All cells were passaged at 60–80% confluence using Gibco TrypLE Express Enzyme.

### Spheroid Culture

MCF7 cells were collected once 80% confluent and counted using an automated cell counter via trypan blue assay. Cell suspensions were diluted using standard cell culture media to 1.2e5 cells mL^−1^. Twenty‐five microliters of cell suspension was pipetted into the center of each well of Nunclon Sphera low‐adhesion round‐bottom 96‐well plates. Following culture for three days, fresh media was added to bring the volume up to 200 µL. Alternatively, at day three spheroids were washed three times with PBS followed by culture in phenol red free DMEM supplemented with Gibco glutaMAX (1%) and charcoal stripped FBS (10%) (white media).

### Bacteria Culture

Prior to chip inoculation, *Lactobacillus plantarum* were grown overnight in Research Products International Corp Lactobacillus De Man, Rogosa, and Sharpe (MRS) broth at 37 °C in a CO_2_ jacketed incubator. All handling for obligate anaerobe, *Blautia coccoides* was conducted in a glove bag purged with ultra‐high purity nitrogen gas and oxygen was removed from culture materials using overnight exposure to AnaeroPack‐Anaero in a sealed chamber. Prior to chip inoculation, *B. coccoides* were grown overnight in BD Bacto Brain Heart Infusion (BHI) broth at 37 °C in an anaerobic jar. For *L. plantarum* and *B. coccoides* coculture, *B. coccoides* were grown in standard BHI or BHI supplemented with 250 µм FITC‐conjugated D‐alanine. For coculture experiments, *L. plantarum* could also be grown in MRS broth in an anaerobic jar. All plating for Colony Forming Unit (CFU) assays were done on BHI agar plates, followed by growth at 37 °C in an anaerobic jar overnight or for several days prior to imaging at ambient conditions.

### Chip Fabrication

Microfluidic chip parts were designed using Autodesk Fusion 360 software prior to milling on a Minitech Computer Numerical Control (CNC) micro‐milling machine. Completed parts were soaked overnight in isopropyl alcohol to remove residual adhesive residue, then dried at 80 °C for 30 min. Chloroform vapor was exposed to channel surfaces by suspending the part over a liquid chloroform bath in a sealed chamber. Chambers were allowed to develop for at least 10 min prior to each use. Upper gut chip channel surfaces were exposed to vapor for 5 min, then assembled with a hydrophilic 0.2 or 1 µm pore size, polycarbonate membrane in a clamping device and baked for 12 min at 105 °C. Channel‐membrane fusions were allowed to rest overnight, then membrane was removed from lower channel port access points using a drop of dichloromethane (DCM). Lower channel pieces and bottom cover slips were exposed to chloroform vapor for 8 and 3 min, respectively, then assembled with channel‐membrane fusions and pressed using a pneumatic heat press at 100 psi and 120 °C for 60 s. Completed chips were stored in a clamping device at room temperature. Tubing interfaces were threaded using a manual thread tapper, their surface exposed to a thin layer of liquid chloroform, then assembled with completed chips. Whole assemblies were then placed in a clamping device to dry overnight. For tissue chips, the same steps were taken with minor modifications. The initial fusion step was conducted with lower channel components and 5 µm polycarbonate membranes, followed by simultaneous fusion to the upper flow channel and bottom coverslip.

### Gut Chip Culture

Assembled polycarbonate gut chips were sterilized by soaking in 70% ethanol for 30 min. Sterilized chips were dried in a tissue culture hood for a few hours or up to overnight. To functionalize polycarbonate membranes for cell seeding, collagen (50 µg mL^−1^ diluted in PBS) was introduced to each channel. Chips were placed in a humid petri dish to limit evaporation, sealed, and left at room temperature overnight. The following day, collagen solution was removed by aspiration, and chips were allowed to dry in a tissue culture hood for half an hour prior to assembly with fluorinated ethylene propylene (FEP) tubing, syringes, and glass reservoirs. Media withdrawn from reservoirs into syringes was incubated in chip channels for 1 h at 37 °C. Alternatively, media was introduced following collagen drying and allowed to incubate at 37 °C overnight followed by tubing assembly and seeding. Caco‐2 cells were collected using either TrypLE or citric saline and counted using an automated cell counter via trypan blue assay. Cell suspensions in standard media were prepared in new reservoirs at a concentration of 3e6 cells mL^−1^. Following media incubation, reservoirs for each upper channel were swapped for those with cell suspension which was gently withdrawn into each chip. Cells were allowed to adhere to the channel surface for 2–2.5 h followed by the replacement of channel media with standard culture media. Flow was then applied to chips at a rate of 100 µL hr^−1^ using a microfluidic syringe pump. Chips were cultured at 37 °C until villi developed, typically six days. Only chips with full cell coverage were used for subsequent experiments.

### Gut Microbiome Chip Bacteria Inoculation

Gut chips were cultured for six days to develop villus‐like structures at which point all reservoirs were exchanged for antibiotic free media, either DMEM supplemented with FBS (10%) or white media. Before use, media for upper channels was depleted of oxygen overnight in an anaerobic jar with Aneropack‐Anaero anaerobic gas generator. On the second or third day of gut chip culture in antibiotic‐free media, bacterial cultures were pelleted (4,000 rcf for 5 min) followed by resuspension in antibiotic‐free cell media. Centrifugation and resuspension were repeated twice to wash the bacteria. Final bacterial suspensions were quantified via NanoDrop and diluted to a final O.D. of 0.5 for single bacteria inoculates or 0.25 each for a two‐species coculture. All media exchanges were conducted in a glove bag purged with ultra‐high purity nitrogen, and final diluted bacteria were prepared in a sealed reservoir. Once sealed, reservoirs were removed from the glove bag and attached in place of upper channel reservoirs for gut chips. Bacteria were gently withdrawn into the chip channel and incubated statically for 1.5 h to allow bacteria to adhere. For longitudinal tracking of two species co‐culture, bacteria were introduced with Biotium NucSpot Live 650 to label all nuclei. Unbound bacteria were subsequently washed away and flow resumed at a rate of 100 µL hr^−1^. For inoculation of *Lactobacillus plantarum* alone, the same protocol was used, except channel reservoirs and bacterial inoculants were prepared in ambient conditions. Following 1 h of culture under flow, some two species co‐culture chips were taken briefly to a microscope to image.

### Tissue Chip Culture

Assembled polycarbonate gut chips were sterilized by soaking in 70% ethanol for 30 min. Sterilized chips were dried in a tissue culture hood for a few hours or up to overnight. Once dry, chips were assembled with FEP tubing, syringes, and collection reservoirs. Spheroids were collected into a tube using a wide bore tip to minimize shearing and excess media was removed. A collagen stock solution in acetic acid was neutralized with sodium hydroxide solution and diluted with 10x PBS to achieve an isotonic solution, prior to mixing with the prepared spheroids. Collagen‐spheroid preparations were allowed to warm at room temperature for 3 min prior to injection into chip culture channels to limit spheroids settling at the bottom of each channel. Chips were incubated at 37 °C for 1 h to allow the collagen to gel, followed by introduction of media into the upper chip channels and layered onto gel injection ports and initiation of flow at a rate of 60 or 100 µL hr^−1^.

### Two‐Photon Laser Scanning Microscopy

A two‐photon microscope consisting of a tunable chameleon Ti:Sapphire laser (100 fs, 80 MHz, Coherent, Santa Clara, CA) on a commercial LSM 880 unit (Zeiss, Santa Clara, CA) was used for imaging. A Plan‐Apochromat 10X/0.45NA air objective (Zeiss) was used to image collagen via Second Harmonic Generation (SHG) as well as fluorescence from live and dead cells via Hoechst 33342 and ethidium homodimer (EthD‐1), respectively. Images (1024 × 1024 pixels) were acquired with the laser tuned at 810 nm for SHG and 740 nm for live and dead cell imaging. Fluorescence emission from Hoechst and EthD‐1 as well as SHG were set to be collected at 450/50, 620/30, and 405/30 nm, respectively.

### Cell Viability Determination

Chips were disassembled from tubing followed by staining with ethidium homodimer (4 µм) and Hoechst 33342 (29 µм) in culture media at 37 °C. Dying chip controls were achieved by preincubation with 70% ethanol. Dying controls in gut chips and tumor chips using more dense collagen (2.7 mg mL^−1^) were additionally stained in the presence of Triton X‐100 (1%). Images containing stained nuclei of either live (Hoechst 33342) or dead (EthD‐1) cells were processed via Volocity (Quorum Technologies, Inc., Puslinch, ON). Cells that had positive staining were thresholded, segmented, and counted with included segmentation tools in Volocity. To determine the viability, the number of live cells were divided by the sum of live and dead. These analyses were performed for each spheroid separately. Statistics were performed using either *t*‐test or one‐way ANOVA.

### Two‐Photon Phosphorescence Lifetime Imaging of Oxygen Sensors (2P‐PLIM)

A two‐photon excitable phosphorescent oxygen reporting micelle complex which contains encapsulated Ru[dpp]^2+^ was fabricated as previously described by Khan et al.^[^ ^51^ ^]^ A gut chip grown for ten days with three days of *B. coccoides* co‐culture was stained with Hoechst for 30 min, followed by introduction of probe solution to the upper, lumen channel. Chip was disassembled from tubing and ports were sealed with cellophane tape in a gas bag to limit introduction of oxygen prior to imaging. 2P‐PLIM of oxygen sensors within the chip was performed using an SPC‐150 fluorescence lifetime imaging (FLIM) module (Becker & Hickl) with an added DDG‐210 pulse generator on the LSM 880 microscope. Excitation of the oxygen sensors occurred at 740 nm with a 3 µs excitation gate for a total of 25.6 µs collection per pixel. Emission from the oxygen sensors was transmitted through a dichroic and further filtered via a 605/70 nm bandpass filter where the light was directed to a BiG detector (Zeiss) which was connected to the photon counting boards. To achieve adequate signal‐to‐noise ratios, 100 frames were collected per image. Data acquisition was performed using Becker & Hickl software (SPCM). The data was further processed via FLIMFit 5.1.1, a custom MATLAB GUI‐based software tool developed by Warren et al., where the phosphorescence lifetime images were generated.^[^ ^52^ ^]^

### Immunostaining

*Gut Chip*: Caco‐2 cells were fixed in‐chip with paraformaldehyde (2%) for 15 min at room temperature. Following fixation, permeabilization and blocking were performed with Triton X‐100 (0.2%) in PBS containing BSA (1%) for 30 min. Fluorophore conjugated primary antibodies were then incubated overnight at 4 °C. The following day, samples were washed 3 × 10 min in PBS. Included in the first wash was 1 µg mL^−1^ DAPI. For high‐resolution imaging, a razor blade was used to carefully separate the bottom two layers of the gut chip from the rest of the chip containing the hydrophilic polycarbonate membrane that the cells were seeded on, the top channel, and the section of the chip containing the media inlet and outlet ports. It was critical not to damage the polycarbonate membrane when using the razor blade to separate the chips. The separated chip was then placed with the membrane containing the cells against a 22 × 40 mm #1.5 coverslip that had a drop of mounting media (Vectashield) to help hold the coverslip in place. Vectashield was then pushed through the media inlet port, ensuring it filled the entire upper channel, and the coverglass was fixed in place using nail polish to seal it to the gut chip.

*Tumor Chip*: Staining of spheroids/organoids in the SCTC and QuadTC chips was accomplished by first pushing paraformaldehyde (4%) and Triton X‐100 (0.1%) into the chip for 60 min at room temperature. Following this fixation, a methanol dehydration/rehydration series was run (25%, 50%, 75%, 95%, 100%, 95%, 75%, 50%, 25%). Samples were then blocked in PBS/Triton X‐100 (0.2%)/BSA (1%) followed by antibody addition in the same solution. Following antibody staining, chips were washed three times in PBS and Triton X‐100 (0.2%), with the first wash containing DAPI at 5 µg mL^−1^. *n* = 1 for each chip type.

*FITC‐D‐Alanine Labeling*: Following one day of co‐culture on‐chip, upper channel reservoirs were exchanged for new reservoirs containing 250 µм FITC‐conjugated D‐alanine or 250 µм FITC‐conjugated D‐alanine with 200 µм pimonidazole. Co‐cultures continued for a second day followed by chip disassembly from tubing. For longitudal study of two species co‐culture, reservoirs were replaced the following morning with freshly deoxygenated media containing Biotium NucSpot Live 650 for 1 h followed by imaging. Chips were fixed in paraformaldehyde (2 or 4%) for 15 min. DAPI staining was conducted at a concentration of 1 µg mL^−1^. For high magnification imaging of bacteria co‐culture, following media collection for CFU assay, fixation with 4% paraformaldehyde, and DAPI staining, chips were broken open and membranes were cut out for direct mounting on slides.

The following antibodies were utilized: #3199 Cell Signaling Technology – E‐Cadherin (24E10)(Alexa Fluor 488) 1:100, #4627 Cell Signaling Technology – Beta‐Catenin (L54E2)(Alexa Fluor 647) 1:100, MA3‐39100‐A555 Invitrogen – ZO‐1 (1A12)(Alexa Fluor555) 1:100, Santa Cruz Biotechnology sc‐515106 AF488 – Mucin‐2 (H‐9) 1:50, Abcam (ab206091) Alexa Fluor 488 Cytokeratin‐18 [EPR1626] 1:500, Santa Cruz Biotechnology sc‐13515 AF647 – HIF1α (28b) 1:50, Jackson ImmunoResearch Laboratories Inc. (715‐475‐150) DyLight 405 Donkey Anti‐Mouse 1:500. Phalloidin Alexa Fluor 555 was used at 0.33 µм, DAPI at 1–5 µg mL^−1^, and D‐Alanine‐FITC at 250 µм.

Antibody/dye labeling images were collected on one of two imaging systems: 1) A Leica THUNDER widefield system on a Leica DMi8 inverted stand equipped with a 10x.32 NA HC PL Fluotar objective or 2) A Zeiss LSM 880 equipped with an AiryScan detector. The standard confocal mode was utilized for most images, however, the AiryScan was utilized for imaging of *Blautia coccoides* labeled with D‐Alanine‐FITC in Figure 3L. Images on this system were acquired using either a 63 × 1.4 NA oil immersion or 40 × 1.2 NA water immersion objective.

### Chip Ab‐/Adsorption Assay

Polycarbonate chips were prepared as stated above. Mock PDMS chips were prepared by casting Sylgard 184 at a ratio of 10:1 into molds which was baked for 4 h. PDMS components were punched for fluid ports and bonded using plasma bonding. Each chip was loaded with rhodamine b (10 µм) and incubated at room temperature for 2 h. Chips were rinsed with PBS, then washed four times for 10 min each in PBS. Images were gathered using a Leica Thunder widefield fluorescence microscope.

### Oxygen Tension Measurement

Gut chips cultured for six days were transitioned to white media. Prior to introduction, inlet media for upper channels was depleted of oxygen overnight in an anaerobic jar with Aneropack‐Anaero anaerobic gas generator. Chips were cultured a further three days. On day 9 of chip culture, OXNANO probes (suspended in distilled water, then diluted in PBS and 10x PBS to maintain osmotic pressure) were introduced to the upper chamber at 100 µL min^−1^ until probe solution reached collection syringes followed by flow at 100 µL hr^−1^ for 10 min. Readings were taken at three points along the channel. Each reading lasted for 2 min taking readings every 5 s. Values at each point were averaged using PyroScience Data Inspector software. Readings were normalized in the software to the proportion of oxygen in ambient air, so output values were multiplied by 20.95% to report %oxygen in the solution. *n* = 4, taken from two independent experiments.

### Permeability Assay

Gut chips were seeded and grown overnight prior to introduction of media bearing cascade blue hydrazide (50 µg mL^−1^) and FITC‐dextran 4 kDa (0.5 mg mL^−1^) in the luminal reservoir. Effluent was collected at day two and three of culture then reservoirs were replaced with dye‐free media. On day six, dye media was again introduced to the luminal reservoirs and cultured overnight. At day seven, media was collected from outlet tubing to eliminate risk of dilution occurring in collection reservoirs. Collected media fluorescence was assessed via plate reader and analyzed using the Emulate P_app_ calculator.

### Bacterial Density Assay

Following two days of culture, gut chips were removed from tubing and transferred to a glove bag purged with ultra‐high purity nitrogen gas alongside deoxygenated BHI agar plates. Media from lumen and circulatory compartments were collected and placed in Eppendorf tubes. Circulatory compartment media and lumen compartment media from control chips without bacteria inoculation were supplemented to reach 100 µL and all was used for even plating. Lumen compartment media from gut chips bearing *L. plantarum* and *B. coccoides* was diluted to 1:1000 and 1:10000 for plating. All plates were transferred to an anaerobic jar with fresh Anaeropak‐Anaero prior to removal from glove bag. Plates were incubated at 37 °C overnight then removed for imaging. CFU were counted manually with FIJI software. To determine bacterial density, dilutions were converted to total CFU mL^−1^, based upon a lumen chamber volume of 40 µL.

### Linked Chip Experiment

Gut chips were grown as described above for five days followed by transition of media reservoirs to white media. At day seven of gut chip culture, five day old MCF7 spheroids that had been transitioned to white media after three days of growth were loaded into QuadTC as described above. Following collagen gelation for 1 h post‐mixing, QuadTC were connected between syringes and gut chip circulatory outlets to receive gut chip circulatory compartment effluent. QuadTC injection ports were layered with white media and circulatory media reservoirs upstream of gut chips were switched to include Estradiol Glow (100nм) or ethanol vehicle control. Flow was reinitiated and continued for 44–48 h. Following two days of linked culture, tubing was removed from all chips. Chips were then stained with Hoechst 33342 for imaging via confocal microscopy or taken straight into RNA extraction for qPCR analysis. Over two independent experiments, two QuadTC chips were treated with vehicle control and three QuadTC were treated with Estradiol Glow.

### Extraction of RNA, cDNA Synthesis, and qPCR

RNA extraction from both the gut and spheroid chips was accomplished using the Norgen Biotek – Single Cell RNA Purification Kit (Cat. 51 800). As per the instructions, 10 µl of 2‐Mercaptoethanol was added to every 1 mL of Buffer RL that was prepared. For RNA extraction from the gut chip, the tubing was removed and the chip rinsed with PBS. Two hundred microliters of RL buffer with 2‐Mercaptoethanol was loaded onto each chip across both channels. Ports were taped, and the whole chip was placed on vortex to agitate intermittently. After 15 min, solution was mixed on chip by pipetting, then transferred to a DNA LoBind tube (Eppendorf 02 243 1021) for further RNA extraction. For the spheroid chip, the chip was opened by splitting the fused layers, thus giving access to the spheroids. Spheroids were collected from each well and placed in 200 µl of RL buffer with 2‐Mercaptoethanol in a DNA LoBind tube. These samples were briefly vortexed and then allowed to incubate at RT for 15 min. After incubation the kit workflow was followed to extract the RNA.

Following RNA isolation, samples were subjected to DNAse treatment via ThermoFisher TURBO DNA‐free kit (Cat. AM1907) and then quantified with ThermoFisher Qubit RNA High Sensitivity (HS) kit (Cat. Q32852). Following quantification, anywhere from 10–150 ng of total RNA was converted to 1st‐strand cDNA utilizing ThermoFisher SuperScript IV VILO Master Mix (Cat. 11 756 050).

Following cDNA synthesis, the cDNA was diluted 1:5 to 100 and 5 µl of cDNA was utilized for each individual qPCR reaction. The qPCR was performed on an Applied Biosystems QuantStudio 6 Flex qPCR system using ThermoFisher PowerUp SYBR Green Master Mix for qPCR in fast cycling mode. The following primer sets were used for SDH (Normalizer) and GREB1 (Estrogen sensitive transcript):

GREB1 Forward:

CAAAGAATAACCTGTTGGCCCTGC

GREB1 Reverse:

GACATGCCTGCGCTCTCATACTTA

SDH Forward:

TGGGAACAAGAGGGCATCTG

SDH Reverse:

CCACCACTGCATCAAATTCATG

The 2^‐(dCt) method^[^ ^53^ ^]^ was used for analysis of all qPCR data.

### Statistical Analysis

Gut chip viability statistical analysis was conducted using one‐way ANOVA with a Bonferroni post hoc following a Brown‐Forsythe test on Graphpad Prism software. Data presented shows [mean+/−SD], *n* = 2–4 gut chips per condition. SCTC spheroid viability statistical analysis was conducted using one‐way ANOVA following a Brown‐Forsythe test on Graphpad Prism software, ****p < 0.0001. Data presented shows [mean+/−SD], *n* = 3 chips and 3 plate spheroids. QuadTC spheroid viability statistical analysis was conducted using Student's *t*‐test following an F‐Test to compare variances on Graphpad Prism software, ***p < 0.005. Data presented shows [mean+/−SD], *n* = 1‐3 spheroids per well, across 4 wells. qPCR analysis was conducted using Welch's t‐test on Graphpad Prism software, *p = 0.013. Data presented shows [mean +/−SEM], *n* = 8–11 wells over two experiments. Analysis of the data with an outlier test (ROUT, Q = 1%) determined that there was a single outlier in the Estradiol Glow dataset [2^‐(dCt) = 1.433] which was then removed before the Welch's t‐test was run.

## Conflict of Interest

The authors declare no conflict of interest.

## Supporting information

Supporting Information

Supplemental Video 1

## Data Availability

The data that support the findings of this study are available from the corresponding author upon reasonable request.
