# Supplementary material for: A Linkable, Polycarbonate Gut Microbiome‐Distal Tumor Chip Platform for Interrogating Cancer Promoting Mechanisms
Source: Adv Sci (Weinh). 2024 Jul 18;11(35):2309220. doi: 10.1002/advs.202309220 (PMC11425222; doi:10.1002/advs.202309220)
Supplement: Supplementary file 1 — Supporting Information [file ADVS-11-2309220-s001.docx]

Supporting Information

A Linkable, Polycarbonate Gut Microbiome-Distal Tumor Chip Platform for Interrogating Cancer Promoting Mechanisms

Danielle S.K. Brasino*, Sean D. Speese, Kevin Schilling, Carolyn E. Schutt, Michelle C. Barton

**Supporting Methods**

*Extracellular Matrix (ECM) Functionalization*

ECM solutions including collagen from rat tail tendon at a concentration of 50µg/mL in either phosphate buffered saline (PBS) or 0.02 м acetic acid or 50µg/mL each of collagen and fibronectin in 1%BSA were prepared and introduced to sterile polycarbonate membranes in chips. Coating was conducted at room temperature or in a tissue culture incubator for either 3 hours or overnight. Following coating, solution was aspirated and membranes allowed to dry for 30 minutes. For fibril deposition imaging, membranes were rinsed with PBS followed by imaging via two-photon microscopy. For cell seeding assays, conducted within a modified chip for static culture, dried membranes were incubated with media for one hour prior to application of cell suspension prepared in conditioned media. Cells were allowed to adhere for 2 hours, 4 hours, 6 hours, or overnight followed by a media wash and incubation prior to imaging. Cells were cultured on membranes for a total of two days followed by DAPI staining at a concentration of 1µg/mL for 30 minutes. Cells were rinsed twice with PBS then imaged via fluorescence microscopy and assessed for monolayer coverage.

*Organoid Culture On-Chip*

Organoids procured from the NCI Patient-Derived Models Repository (Patient ID 487391, Specimen ID 300-R) were prepared following protocols from the National Cancer Institute Patient-Derived Models Repository. Briefly, organoids were cultured in a complete media composed of Advanced DMEM/F12 with HEPES, GlutaMAX, Primocin, and L-WRN cell conditioned media further supplemented with N-acetylcysteine, nicotinamide, B-27, N-2 and Y-27632 dihydrochloride. To passage organoids, a simplified wash media (composed of advanced DMEM/F12, HEPES, GlutaMAX, Primocin and FBS) was supplemented with Dispase II and added to organoid cultures. Following incubation at 37^o^C for 1.5-2 hours, cultures were dissociated by pipetting followed by neutralization in more simplified wash media and centrifugation. Supernatant was removed, wash media added and mixed with organoids, then centrifuged again. After removing supernatant, organoids were further dissociated by repeated pipetting then all media was removed. Remaining organoids were mixed with Reduced Growth Factor BME Type 2 on ice and injected into sterilized SCTC. Chips were incubated at 37^o^C for 15 minutes to allow BME2 to set, followed by introduction of media and initiation of flow at a rate of 10.5 µL/hr.

**Supporting Data**

**
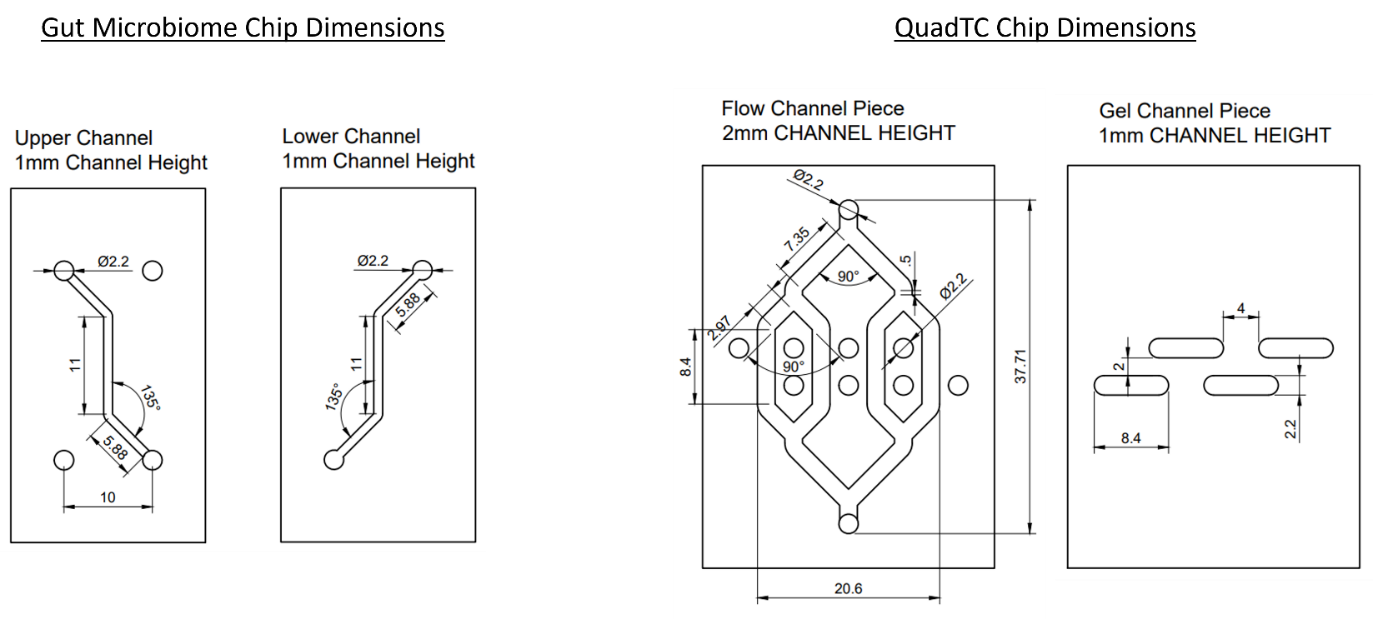
**

**Figure S1.** Schematics of manufacturing dimensions for gut microbiome chip and QuadTC chip channel components.


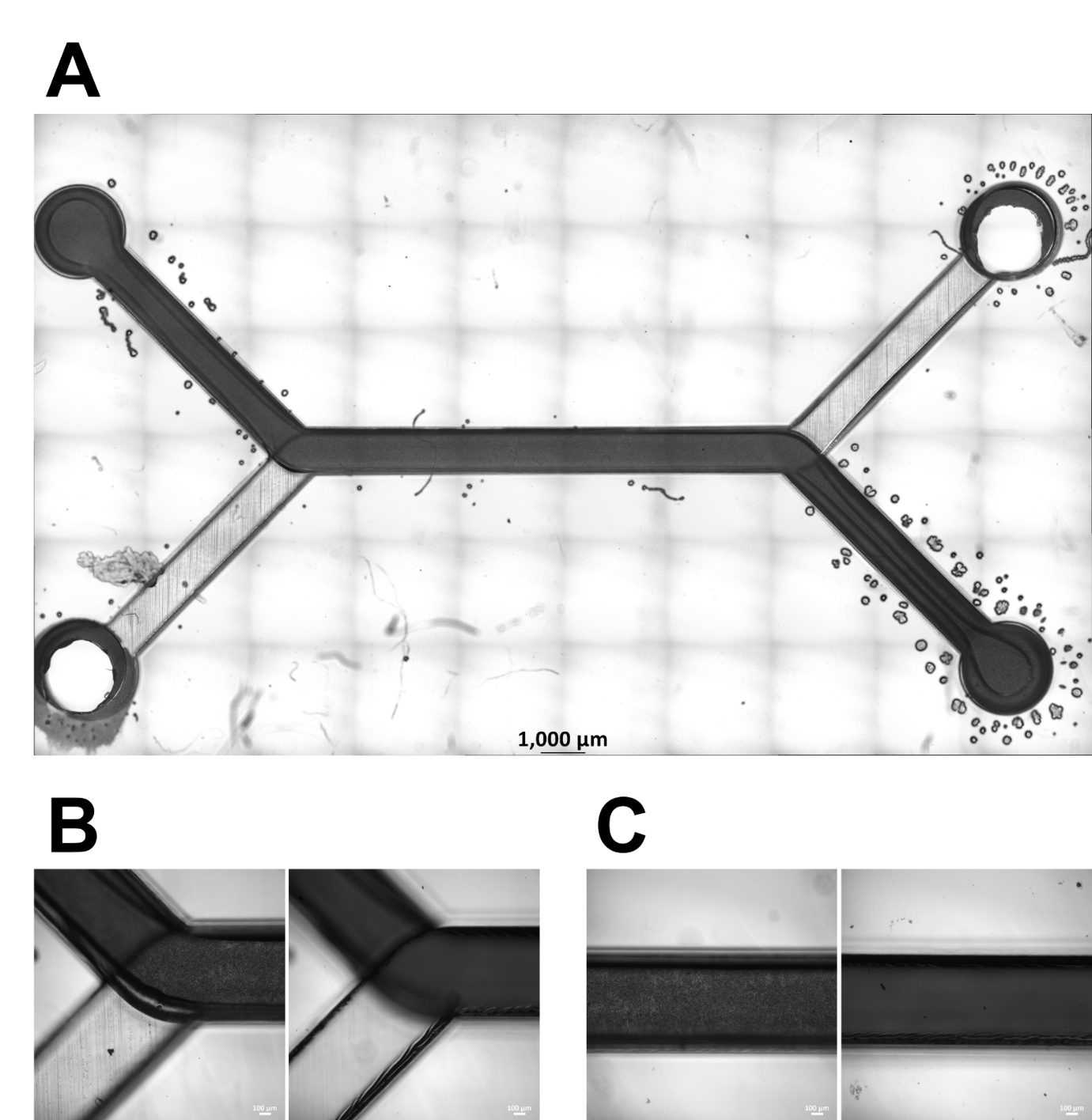


**Figure S2.** A) Stitched image collected via brightfield microscopy depicting minimal deformation of gut microbiome chip channels during the fusion process. Lumen channel extremities in the non-overlapping regions show membrane warping due to contact and fusing with lower channel plastic. B) Brightfield image of a gut chip at the end of the overlapped channel region, showing two z-planes. C) Brightfield image of a gut chip in the overlapping region, showing two z-planes.


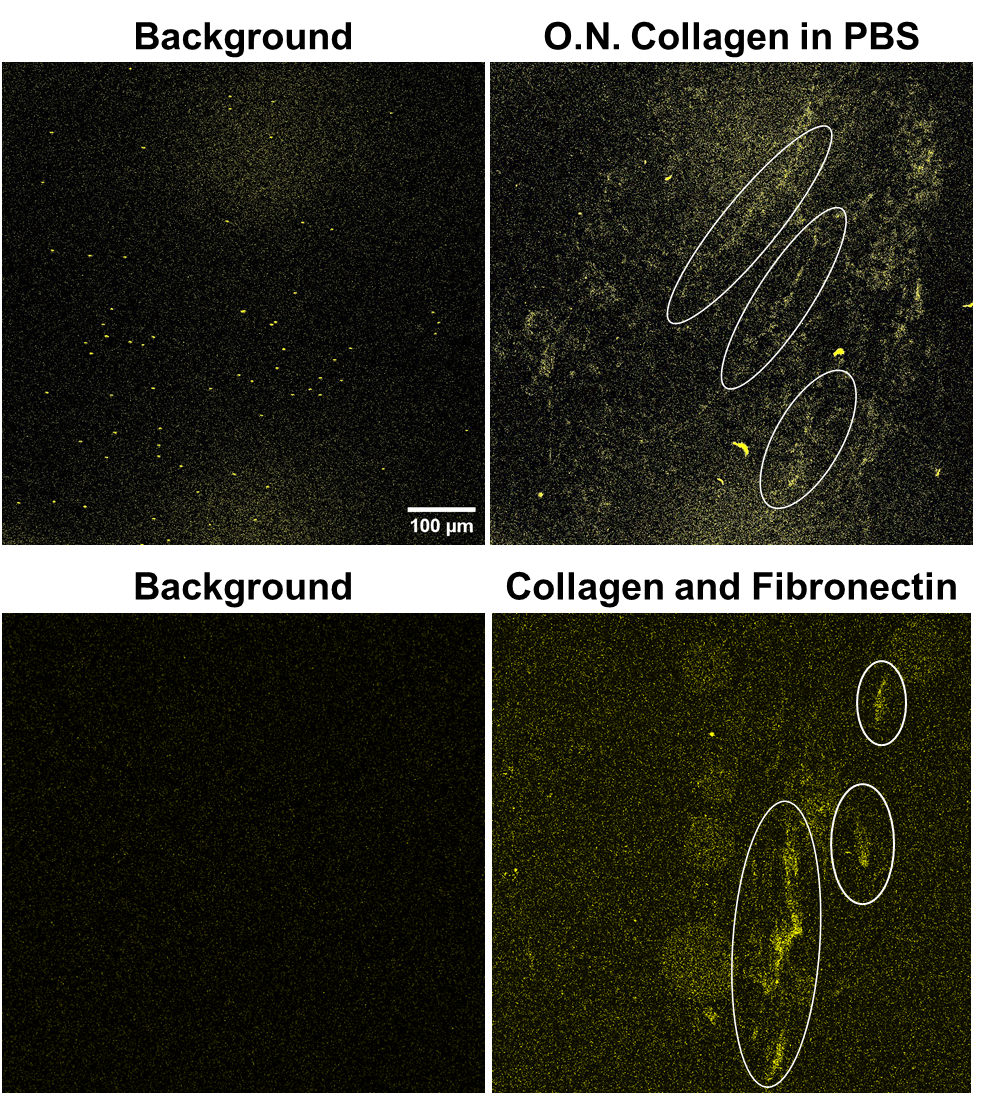


**Figure S3.** Imaging of collagen fibril deposition *via* two-photon microscopy. Images show deposition conducted overnight (O.N.) in PBS or deposition conducted using a collagen and fibronectin mixture. Examples of collagen fibrils are noted by white ovals.


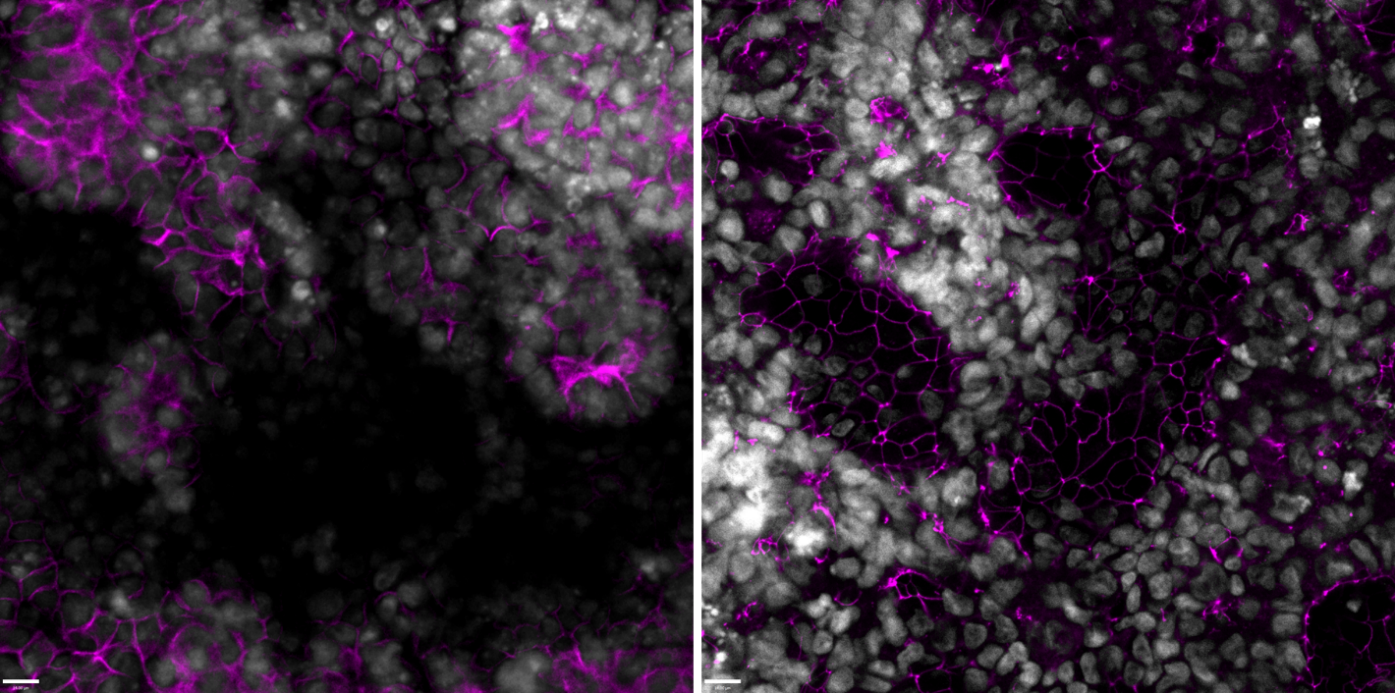


**Figure S4.** Confocal microscopy of gut chips stained for beta catenin(left) and ZO1 (right) showing a larger field of view of images in the text. Scale bar denotes 14µm.


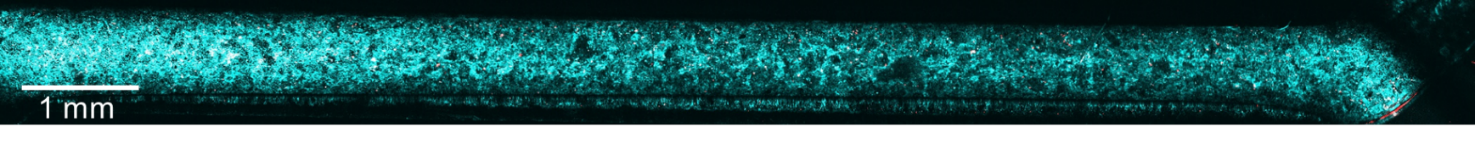


**Figure S5.** Two-photon microscopy image of gut epithelium after two days of co-culture with *Blautia coccoides,* staining for live cells (blue) and dead cells (red).


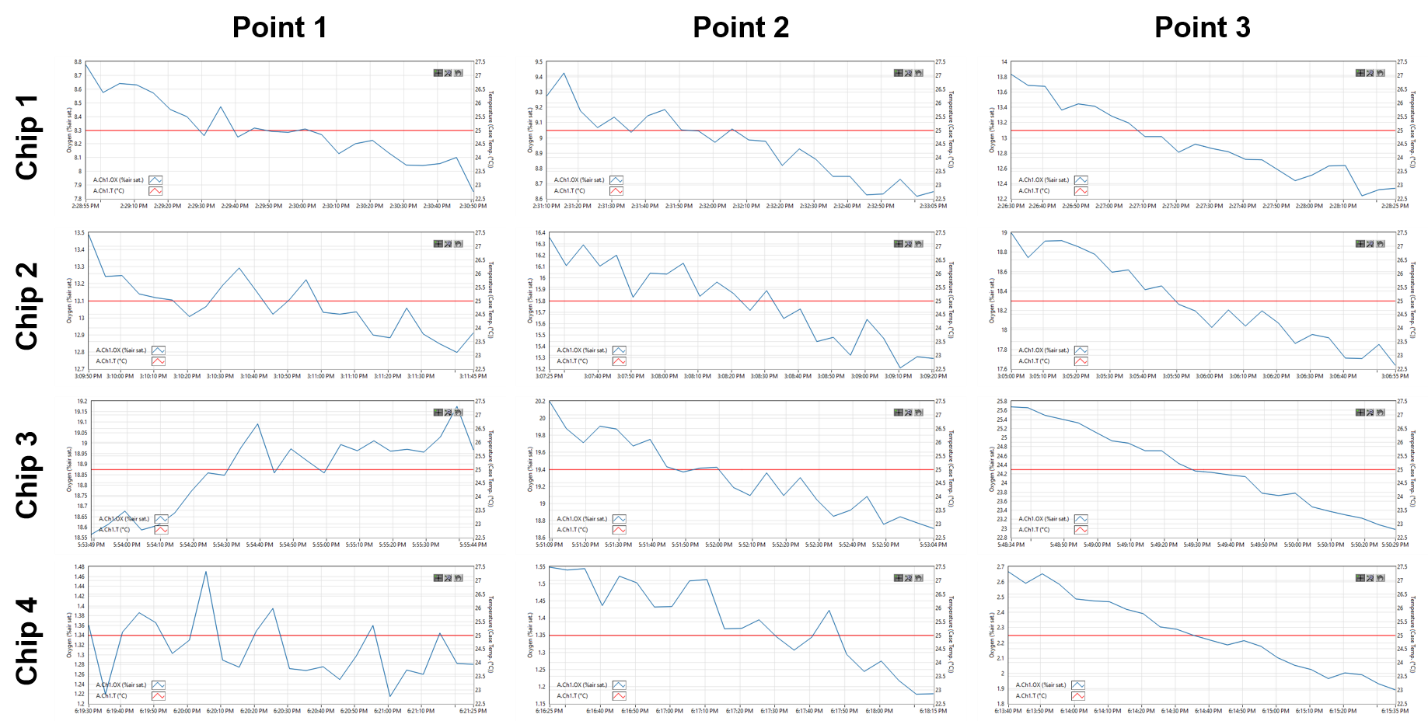


**Figure S6.** Readings taken from nine day-old gut chips using PyroScience’s OXNANO bulk oxygen sensor flowing through the deoxygenated lumen chamber.


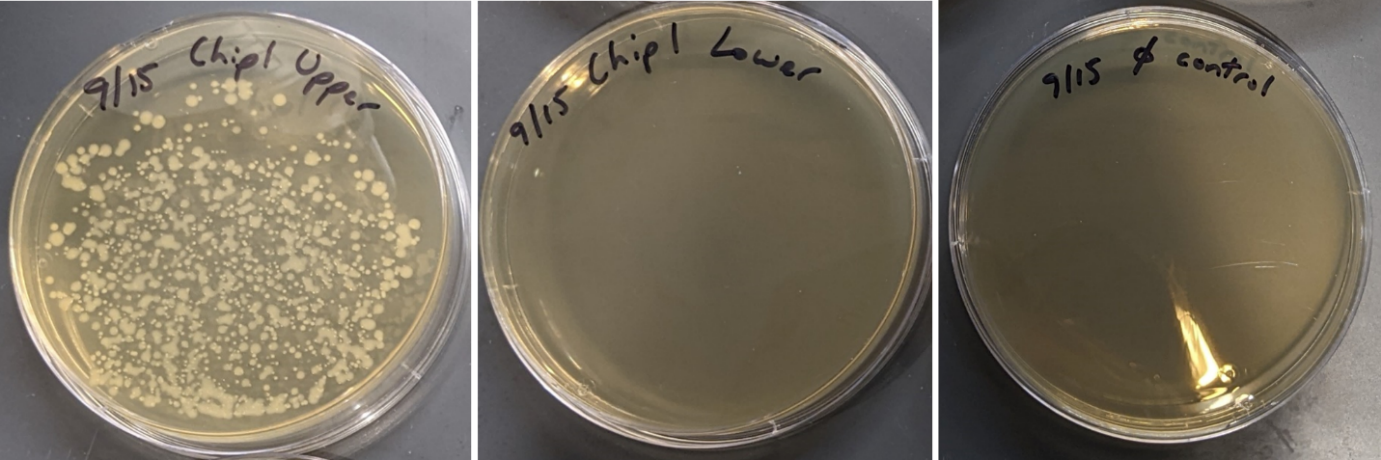


**Figure S7.** Plating media collected from the upper lumen compartment and the lower circulatory compartment of chips assembled using 0.2 µm pore membranes following two days of co-culture between Caco-2 epithelium and obligate anaerobe *B. coccoides* demonstrate bacteria maintain viability in the polycarbonate gut microbiome chip.


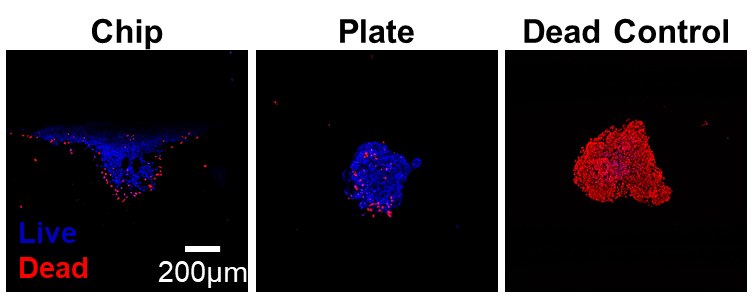


**Figure S8.** MCF7 spheroids cultured for one week in low adhesion u-bottom plates were loaded into SCTC with 1.45mg/mL collagen. Following culture at 60 µL/hr flow for two days, three chips and three spheroids kept in plate culture were assessed for viability via staining with Hoechst for live cells (blue) and ethidium homodimer for dead cells (red). Chip culture did not reduce spheroid viability.


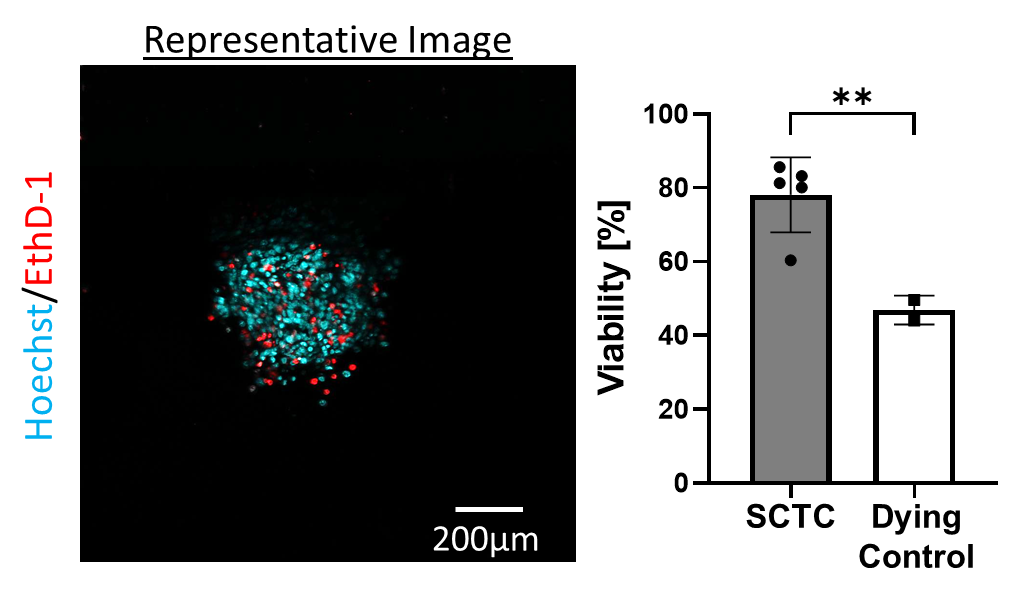


**Figure S9.** MCF7 spheroids cultured for five days in low adhesion u-bottom plates were loaded into SCTC with 2.7mg/mL collagen. Following culture at 100 µL/hr flow for three days, three chips were assessed for viability with one dying spheroid control via staining with Hoechst for live cells (blue) and ethidium homodimer for dead cells (red). Spheroids maintained high viability when cultured on chip. (**, p<0.01, Student’s t-Test, error bars denote standard deviation).


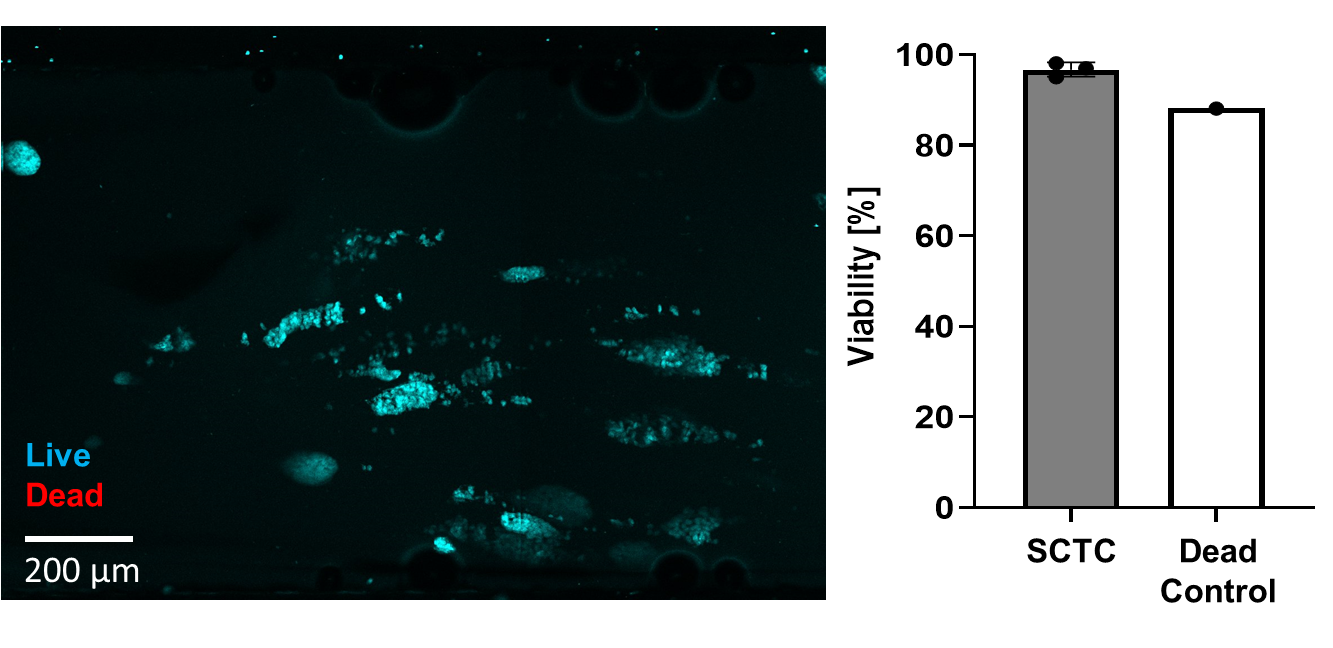


**Figure S10.** Kidney cancer patient-derived organoids were passaged and loaded into the SCTC in BME2. After letting the hydrogel set, chips were cultured with constant flow at 10.5 µL/hr for one week. Three chips were assessed for viability by staining with Hoechst for live cells (blue) and ethidium homodimer for dead cells (red). A fourth chip was treated as dying organoid control. Each chip value was determined by averaging values for multiple organoids. Organoids maintain high viability on-chip. Error bars denote standard deviation.


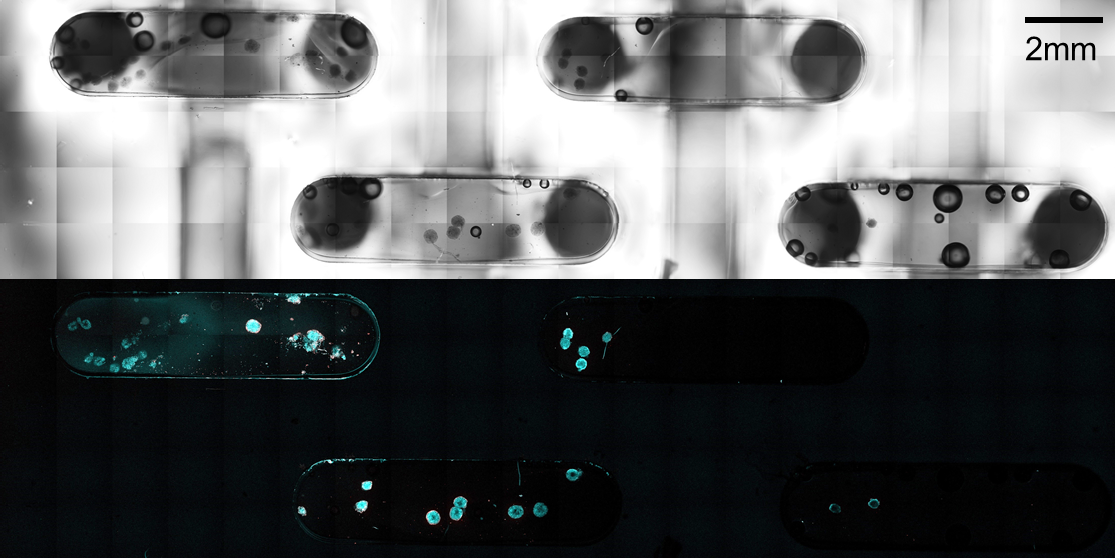


**Figure S11.** MCF7 spheroids cultured for five days in low adhesion u-bottom plates with transition to white media at day three were loaded into QuadTC in 2.7mg/mL collagen. Following culture under flow for two days, chip was assessed for viability via staining with Hoechst for live cells (blue) and ethidium homodimer for dead cells (red). Image shows stitched panels in brightfield (top) and fluorescence microscopy (bottom) to cover all four culture wells. Spheroids maintained high viability when cultured on-chip.


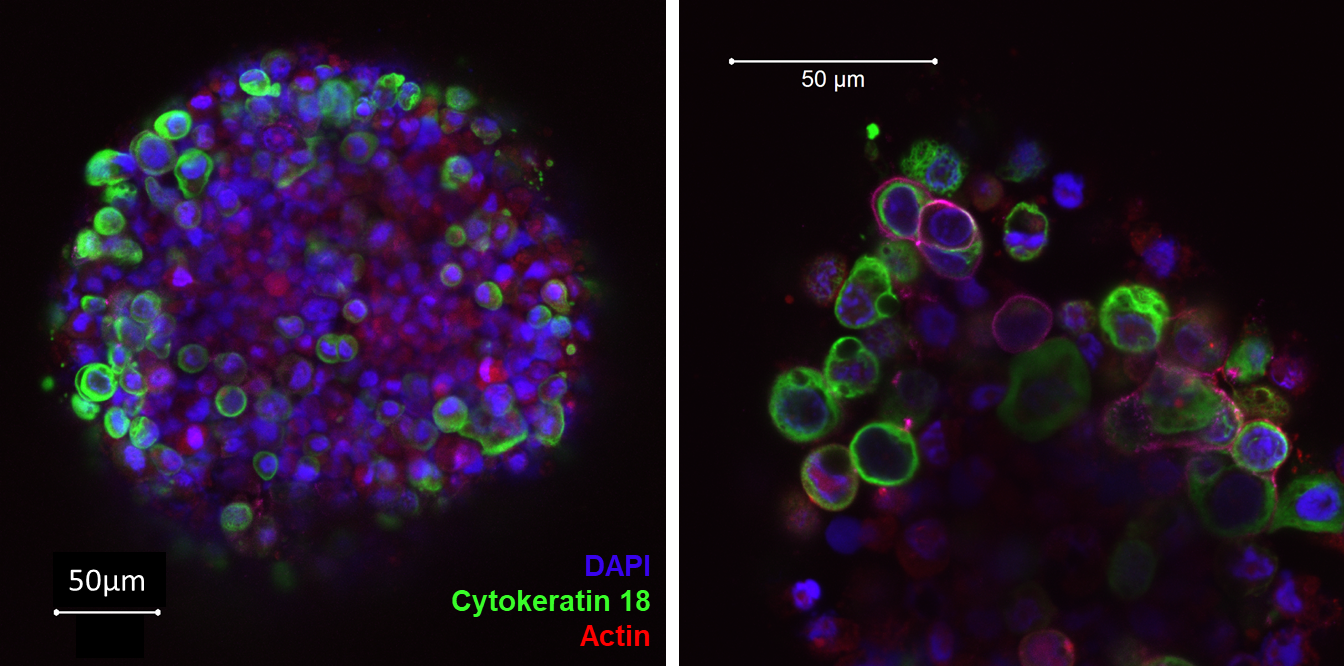


**Figure S12.** High resolution on-chip imaging in SCTC**.** MCF7 spheroids cultured for one week in low adhesion u-bottom plates were loaded into SCTC in 2.7mg/mL collagen. Following culture under flow at 60µL/hr for two days the chip was simultaneously fixed and permeabilized. Chip was dehydrated with methanol then rehydrated in PBS followed by blocking and staining using a tissue processing microwave. n=1

**Video 1.** Gut chips culturing Caco-2 cells for one week were inoculated with *Blautia coccoides.* After one day of co-culture, inlet media was supplemented with 250 µм FITC-D-alanine. Following another day of co-culture to allow incorporation of D-alanine into bacterial cell walls, chips were rinsed and fixed. Video depicts a z-stack of FITC-D-alanine labelled *B. coccoides* (green) growing at the surface of a Caco-2 villus-like structure, Caco-2 cell nuclei labelled with DAPI (blue).
